# Single Nucleotide Polymorphisms of Interleukins and Toll-like Receptors and Neuroimaging Results in Newborns with Congenital HCMV Infection

**DOI:** 10.3390/v13091783

**Published:** 2021-09-07

**Authors:** Justyna Czech-Kowalska, Dominika Jedlińska-Pijanowska, Agata K. Pleskaczyńska, Anna Niezgoda, Kinga Gradowska, Aleksandra Pietrzyk, Elżbieta Jurkiewicz, Maciej Jaworski, Beata Kasztelewicz

**Affiliations:** 1 Neonatal Intensive Care Unit, The Children’s Memorial Health Institute, 04-730 Warsaw, Poland; d.jedlinska-pijanowska@ipczd.pl (D.J.-P.); a.pleskaczynska@ipczd.pl (A.K.P.); a.niezgoda@ipczd.pl (A.N.); k.gradowska@ipczd.pl (K.G.); a.pietrzyk@ipczd.pl (A.P.); 2 Department of Diagnostic Imaging, The Children’s Memorial Health Institute, 04-730 Warsaw, Poland; e.jurkiewicz@ipczd.pl; 3 Department of Biochemistry, Radioimmunology and Experimental Medicine, The Children’s Memorial Health Institute, 04-730 Warsaw, Poland; m.jaworski@ipczd.pl; 4 Department of Clinical Microbiology and Immunology, The Children’s Memorial Health Institute, 04-730 Warsaw, Poland; b.kasztelewicz@ipczd.pl

**Keywords:** human cytomegalovirus (HCMV), congenital CMV infection, central nervous system, interleukins (IL1B; ILB28), toll-like receptor (TLR), single nucleotide polymorphism (SNP), neuroimaging, MRI

## Abstract

Congenital cytomegalovirus infection (cCMV) is the most common intrauterine infection with central nervous system (CNS) involvement. There is limited data on the associations between Single Nucleotide Polymorphisms (SNPs) in genes involving the first-line defense mechanism and the risk of CNS damage during cCMV. We investigated the associations between neuroimaging findings and SNPs in genes encoding the following cytokines and cytokine receptors in 92 infants with cCMV: interleukins (IL1B rs16944, IL12B rs3212227, IL28B rs12979860), C-C motif chemokine ligand 2 (CCL2 rs1024611), dendritic cell-specific intercellular adhesion grabbing non-integrin (DC-SIGN rs735240), Toll-like receptors (TLR2 rs5743708, TLR4 rs4986791, TLR9 rs352140). The SNP of IL1B rs16944 (G/A) was associated with a reduced risk of ventriculomegaly on MRI (OR = 0.46, 95% CI, 0.22–0.95; *p* = 0.03) and cUS (OR = 0.38, 95% CI, 0.0–0.93; *p* = 0.034). Infants carrying heterozygous (T/C) genotype at IL28B rs12979860 had an increased risk of cystic lesions on cUS (OR = 3.31, 95% CI, 1.37–8.01; *p* = 0.0064) and MRI (OR = 4.97, 95% CI, 1.84–13.43; *p* = 0.001), and an increased risk of ventriculomegaly on MRI (OR = 2.46, 95% CI, 1.03–5.90; *p* = 0.04). No other associations between genotyped SNPs and neuroimaging results were found. This is the first study demonstrating new associations between SNPs of IL1B and IL28B and abnormal neuroimaging in infants with cCMV.

## 1. Introduction

Congenital human cytomegalovirus infection (cCMV) is the most common intrauterine infection. Human cytomegalovirus (HCMV) transmitted through placenta to immature fetus can lead to serious central nervous system (CNS) damage and sensorineural hearing loss (SNHL), with a long term and unfavorable sequelae [1,2]. The neurotropism of HCMV is evident from the predominance of CNS abnormalities observed in fetuses and neonates with cCMV [1,3,4]. HCMV may infect a wide spectrum of cell types, including neurons, astrocytes, radial glia, and endothelial cells, and may disrupt neuronal proliferation, migration, and cortical cell organization [1,5]. A higher number of infected cytomegalic cells, and a loss of germinal and radial glial cells in periventricular regions, is detected in the brains of infants with severe cCMV [6]. The most profound changes are located in the cortex, white matter, germinal matrix, grey matter, ependyma, and leptomeninges of affected fetuses; the brainstem and cerebellum are less often involved [4]. Neuroimaging plays an important role in a diagnostic work-up if cCMV is suspected. While an ultrasound (US) is the method of choice for fetal and neonatal imaging, magnetic resonance imaging (MRI) has an established added value in the detection of fetal and neonatal brain anomalies, including white matter signal abnormalities, cortical defects (e.g., polymicrogyria), and cerebellar hypoplasia/dysplasia [3,7]. Abnormal neuroimaging results may predict an increased risk of adverse neurodevelopmental sequalae with a high negative predictive value for fetal MRI findings [7,8,9,10].

There is limited data on risk factors and reasons for the wide spectrum of CNS damage due to cCMV. Undisputedly, a massive and widespread viral infection in the early stage of brain development leads to the most severe brain damage [4,11]. One of the important factors is HCMV strain virulence, which is dependent on at least genetic variability in genes, which encodes the envelope glycoprotein N (gN). This protein is essential for the virus’ attachment to the host cell, viral entry, and subsequent viral spread [12]. A significant association between gN-2 or gN-4 genotypes and symptoms of cCMV, including a neurological sequelae, was reported. On the other hand, gN-1 and gN-3a genotypes represent less pathogenic HCMV strains and reduce the risk of an unfavorable outcome in infants with cCMV [13,14].

According to host genetic studies, we can gain insight into biological mechanisms involved in HCMV pathogenesis [15]. The first-line host defense mechanism against pathogens (including HCMV) is an innate immune response. Toll-like receptors (TLR-2, TLR-4, TLR-9) and dendritic cell-specific intercellular adhesion grabbing non-integrin (DC-SIGN) are important in a process of HCMV recognition and triggering inflammatory cytokines [16,17,18,19,20,21]. Interleukins (ILs) such as IL1B, IL12B, and IL28B play a proinflammatory and immunomodulatory role [22,23,24,25], whereas C-C motif chemokine ligand 2(CCL 2) and a monocyte chemotactic protein–1 (MCP-1) are important in immune cells’ differentiation and migration [26].

Single Nucleotide Polymorphisms (SNPs) in genes of innate immunity may alter their expression and influence on the immune response against HCMV, especially during the most vulnerable period of pregnancy and fetal and neonatal life. So far, SNPs has been quite extensively studied in the context of acquired HCMV infection in organ transplant patients and stem cells recipients [16,23,27,28,29,30,31]. However, there are still only a few studies linking the SNPs of ILs or TLRs to a predisposition to cCMV and symptoms of the disease in newborn infants [16,17,20,22,25,32,33]. Based on the abovementioned reports, we selected for this study SNPs of eight candidate genes which we considered to be potentially important in cCMV infection. As far as ILs are concerned, SNPs of IL1B (rs16944 and rs3954) have been shown to be associated with cCMV infection and maternal HCMV infection [22,34]. We decided to select IL1B SNPs (rs16944) because of a previously reported association between a rare T/T genotype of IL1B rs16944 polymorphism and an increased risk of cCMV infection in a Polish population [22]. The C allele in IL12B rs3212227 was also related to an increased risk of HCMV reactivation [23]. While T allele in the SNP of IL28B (rs12979860)—with a lower incidence of HCMV infections among kidney transplant patients [30]—was selected for the current analysis. We also chose the SNP of CCL2 because of a previously reported association between CCL2 (rs102461) and sensorineural hearing loss in Polish infants with cCMV [22]. Additionally, the SNP of DC-SIGN (rs735240 G > A) was also incorporated into the study due to its reported role in acquired HCMV infection in European populations (Spain/Germany) after kidney or allogenic stem cell transplantation [15]. The role of TLR SNPs in congenital and acquired HCMV infection was reported for TLR2, TLR3, TLR4, TLR7, and TLR9 SNPs in various populations [16,17,20,29,32,35,36]. Two TLR2 SNPs (rs3804100: T > C and rs1898830: A > G) have been shown in Japanese infants to have some association with congenital CMV infection, while the TLR2 SNP (rs3804100 GG genotype) has shown a protection against intrauterine transmission of HCMV in an Israeli cohort [37]. Finally, we selected TLR2 sr5743708 due to a previously reported correlation between the G/A heterozygotic status at TLR2 rs5743708 and cCMV infection in Polish fetuses and newborns [20]. Another SNP of the TLR4 variant (rs4986791) was selected because this variant diminished the risk of CMV infection in an adult Polish population, although had no effect in a pediatric population [38]. As far as TLR9 SNPs are concerned, we selected SNP (rs352140), which was involved in maternal HCMV infection in Polish pregnant women and cCMV infection in Polish newborn infants [17,39]. Both GG homozygous and GA heterozygous variants in TLR9 (rs352140 G > A) were found to be significantly associated with a decreased occurrence in HCMV infection among Polish pregnant women [39]. TLR9 SNP (rs 352140 G > A) seems to be the major polymorphism, contributing to HCMV infection in pregnant women [39]. Not all ILs and TLRs SNPs that have been shown to play a role in HCMV infection were included in this study for various reasons, however ethnic differences were the most important.

The objective of the study was to evaluate the association between SNPs in eight candidate genes with a plausible contribution to cCMV disease (including IL1B rs16944, IL12B rs3212227, IL28B rs12979860, CCL2 rs1024611, DC-SIGN rs735240, TLR2 rs5743708, TLR4 rs4986791, TLR9 rs352140) and neuroimaging results in newborns with cCMV.

## 2. Materials and Methods

### 2.1. Study Population

We prospectively enrolled Caucasian neonates with confirmed cCMV admitted into the Neonatal Intensive Care Unit of The Children’s Memorial Health Institute in Warsaw between 2016 and 2019 [33]. Inclusion criteria were positive real-time PCR DNA CMV in urine ≤ 21st day of life, and parental consent. Exclusion criteria were: multiple congenital anomalies, severe course of bacterial septic disease, other TORCH infections (e.g., congenital toxoplasmosis), lack of parental consent.

### 2.2. *Methods*

All neonates with cCMV infection underwent thorough physical examination, ophthalmologic and hearing evaluation, and newborn hearing screening tests (Otoacoustic Emission (OAE) and Auditory Brainstem Response (ABR)). SNHL was defined as air conduction thresholds > 20 dB HL on the ABR. Laboratory blood tests to assess bone marrow and hepatobiliary function were performed. The following definitions for laboratory abnormalities were used: elevated direct bilirubin >1 mg/dL for cholestasis, elevated level of aspartate transaminase (ASPAT) *>* 84 U/L and/or elevated level of alanine aminotransferase (ALTAT) *>* 60 U/L for hepatitis, thrombocytopenia below 100 K/μL, and neutropenia below 1000 K/μL for bone marrow involvement. As a part of routine work-up, blood and urine samples were collected for qualitative and quantitative real-time PCR DNA HCMV assessment, as described previously [22,33]. The same blood samples were used for the SNPs’ genotyping test. Lumbar puncture was a part of a routine clinical procedure in the work-up of infants with cCMV and CNS involvement in our unit. Lumbar puncture was performed after parental informed consent had been obtained. Neonatal cerebrospinal fluid (CSF) evaluation included a standard cytochemical analysis and real-time PCR DNA CMV assessment. An elevated CSF protein concentration was defined as >115 mg/dL [40]. A symptomatic cCMV was diagnosed when at least one of the following symptoms of CNS involvement was present: microcephaly, senso-neuronal hearing loss (SNHL), chorioretinitis, abnormal neuroimaging, or at least three abnormalities of the hepatobiliary and reticuloendothelial system.

#### 2.2.1. Neuroimaging

Neuroimaging was performed routinely at the baseline evaluation during hospital stay. Cranial US (GE Helthcare, Vivid 7 dimension ’06 or Philips Affiniti 50G, transducer C8-5 and/or L12-4) was performed by qualified neonatologist. All brain MR examinations were performed using a 1.5 T scanner (Avanto fit, Siemens, Erlangen, Germany) during natural sleep after feeding. Only pacifier and soft oral sedatives were used. A specific pediatric 16-channel head coil or 20-channel head and neck coil were used, and the following imaging protocol was performed: TSE T2-weighted images in axial (TR/TE, 6000/181 ms), coronal (TR/TE, 6000/181 ms), sagittal (TR/TE, 6000/216 ms) planes, axial TSE tir T1-weighted images (TR/TE/TI, 5580/48/350 ms), axial SE T1-weighted images (TR/TE, 690/13 ms), axial dark-fluid fs T2-weighted images (TR/TE/TI, 9000/108/2500 ms), fl3D-iso T1-weighted images (TR/TE, 9.2/4.8 ms), diffusion-weighted imaging—DWI (TR/TE, 5900,87 ms) with b-values of 0/500/1000 mm²/s, susceptibility-weighted imaging—SWI (TR/TE, 49/40 ms). Slice thickness from 1 to 3 mm. Field of view—FOV 109- to 180 mm × 170- to 200 mm. The heartrate and transcutaneous oxygen saturation were monitored during examination by a pulse oximeter. MRI scans were assessed by experienced radiologists. Abnormal neuroimaging findings were defined as ventricular dilatation, cystic lesions (parafrontal, caudothalamic and temporal germinolytic cysts, and peri/intraventricular cyst), periventricular calcifications, white matter abnormalities, cortical migration defect (e.g., polymicrogyria, pachygyria, lissencephaly), cerebellar hypoplasia, dysgenesis of corpus callosum and abnormal myelination.

#### 2.2.2. Determination of SNP Genotypes

SNPs genotyping was performed using genomic DNA extracted from whole blood collected for the detection of HCMV. Briefly, TaqMan SNP Predesigned Genotyping Assays (Applied Biosystems, Inc., Foster City, CA, USA) were applied for IL1B rs16944, IL12B rs3212227, IL28B rs12979860, CCL2 rs1024611, DC-SIGN rs735240, TLR2 rs5743708, TLR4 rs4986791, TLR9 rs352140 polymorphisms. The allelic discrimination was performed on the 7500 Real-time PCR System (Applied Biosystems, Inc., Foster City, CA, USA) according to the manufacturer’s instructions. A blinded duplicated genotyping of 10 random study samples demonstrated 100% concordance.

The minor allele frequencies (MAF) for the European population of the analyzed SNPs are presented in Table 1 (available from: NCBI ALFA project; Release Version: 20201027095038; https://www.ncbi.nlm.nih.gov/snp/docs/gsr/alfa/ (accessed on 16 August 2021)).

#### 2.2.3. Statistical Analyses

The Shapiro–Wilk test was used for the assessment of normality of the distribution of analyzed data. Departures from Gaussian distribution were ascertained. Thus, data were presented as median and interquartile ranges (IQR), and a non-parametric Mann–Whitney test was used because data was not normally distributed. Counts were presented as numbers and percentages, and a Chi-squared test was used. In the cases where the expected number of observations was less than 5, Fisher’s exact test was used. Deviation from the Hardy–Weinberg equilibrium (HWE) for each SNP was evaluated by using the Chi-square test. The association between the SNP genotype and the congenital abnormal neuroimaging findings in US and MRI was analyzed by co-dominant, dominant, recessive, over-dominant, and additive models. The Akaike information criteria (AIC) was used to select the best fitted model. The model with the lowest AIC value was the best model. Odds ratios (ORs) with 95% confidence intervals (CIs) for each model were calculated using logistic regression [31]. The association and HWE analyses were performed using SNP Stats software [41]. Otherwise, statistical analyses were performed using STATISTICA software version 10 (StatSoft Polska, Krakow, Poland). No correction was made for multiple testing; *p*-value < 0.05 was considered significant.

## 3. Results

There were 92 newborn infants with confirmed cCMV included. The median (IQR) gestational age was 38 weeks (IQR: 37–39), birth weight was 2755 g (IQR: 2170–3200), and head circumference at NICU admission was 34.1cm (IQR: 32.0–35.5). Among the study population, 23 infants (25%) were born prematurely, 42% of the infants by primipara and 49% of infants by cesarean section. There were 73 (79%) symptomatic infants treated with valganciclovir and 19 infants with asymptomatic cCMV without treatment. Symptomatic infants had a lower birth weight (median 2685 g (IQR: 2150–3140 g) vs. 3050 g (IQR: 2680–3630 g); *p* < 0.05) and were born more often prematurely (19 (26.03%) vs. 4 (21.05%); *p* < 0.05) than asymptomatic infants, respectively. The most frequent symptoms of cCMV were abnormal muscles tone (59%), abnormal ABR in at least one ear (35%), thrombocytopenia (30%), IUGR (28%), and microcephaly (24%) (Table 2).

### 3.1. Neuroimaging Findings

A total of 92 infants had a brain US followed by MRI (the MRI result was not available for assessment in five infants due to technical reasons, namely substantial movement artifacts). Neuroimaging findings in newborn infants with cCMV are presented in Table 3. The most frequent findings on neuroimaging were cystic lesions on cUS and abnormal white matter on MRI (Table 3). Among nineteen infants with normal cUS, seventeen underwent MRI. As a result, eight infants had abnormal MRI: six infants only had abnormal white matter, which is normally not seen on cUS, one infant had ventricular dilatation, and another one also had cortical migration defects detected.

### 3.2. Frequencies of the SNPs Genotypes in the Study Population

Eight selected SNPs were genotyped successfully in the study population [33]. The genotypic frequencies of SNPs for the overall study population are shown in Table 4**.** The observed genotypes distribution in the study population did not significantly deviate from the expected counts, according to HWE for all SNPs (*p* > 0.05).

### 3.3. Associations between Selected Gene SNPs and cUS Results in Infants with cCMV

Regarding SNPs genotype distribution and our findings on cUSG, associations with cystic lesions and ventricular dilatation were found. In particular, a significantly higher proportion of individuals carrying heterozygous (C/T) genotype at IL28B rs 12979860 was observed among infants with cCMV and cystic lesions compared with those without cystic lesion (54% vs. 26.2%; OR = 3.31, 95%CI, 1.37–8.01; *p* = 0.0064) in the most fitted genetic model, being the overdominant model (Table 5).

Infants carrying heterozygous (G/A) or homozygous for minor allele (A/A) genotype of IL1B rs16944 had a reduced risk of ventricular dilatation on cUS compared with those carrying homozygous for major allele (G/G) genotype (OR = 0.38, 95% CI, 0–0.93; *p* = 0.034) in the best fitted dominant model (Table 6). No other associations between genotyped SNPs and cUS results (lenticulostriate vasculophaty, periventricular echogenicity, calcifications) were found in infants with cCMV (Tables S1–S3).

### 3.4. Associations between Selected Gene SNPs and MRI Results

Considering the SNPs’ genotype distribution and findings on MRI, associations with cystic lesions and ventricular dilatation were found. The SNP of IL1B rs16944 was associated with a decreased risk of ventriculomegaly (OR= 0.46, 95%CI, 0.22–0.95; *p* = 0.03) in the best fitted log-additive model (Table 7). In addition, a significantly higher proportion of individuals carrying heterozygous (C/T) genotype at IL28B rs 12979860 was observed among infants with cCMV and ventriculomegaly compared with those with normal ventricular size (53.5% vs. 31.8%; OR = 2.46, 95%CI, 1.03–5.90; *p* = 0.04) in the most fitted genetic model, being the overdominant model (Table 6). The SNP of IL28B rs 12979860 was also associated with cystic lesions on MRI. A significantly higher proportion of individuals carrying heterozygous (C/T) genotype at IL28B rs 12979860 was observed among infants with cCMV and cystic lesions compared with those without cystic lesion (69.2% vs. 31.1%; OR = 4.97, 95%CI, 1.84–13.43; *p* = 0.001) in the most fitted genetic model, being the overdominant model (Table 8). No other associations between the examined SNPs and the MRI findings were found, including abnormal myelination, abnormal white matter, calcifications, and cortical migration defects in infants with cCMV (Tables S4–S7).

## 4. Discussion

This prospective study of the cohort of 92 Caucasian newborn infants with cCMV investigated the association between eight SNPs in genes encoding cytokines and cytokine receptors (IL1B rs16944, IL12B rs3212227, IL28B rs12979860, CCL2 rs1024611, DC-SIGN rs735240, TLR2 rs5743708, TLR4 rs4986791, and TLR9 rs352140) with neonatal neuroimaging findings. The results of our study demonstrated novel associations between the SNPs of IL28B and IL1B and the neonatal neuroimaging findings in cCMV. Infants carrying the C/T genotype of IL28B rs12979860 had a three to four times increased risk of cystic lesions on cUS or MRI, and more than a two-fold increased risk of ventricular dilatation diagnosed by MRI. On the other hand, the SNP of IL1B rs16944 (G/A) was associated with more than a 50% reduction in risk of ventricular dilatation diagnosed by MRI or cUS. We found no association between SNPs in the other tested genes of ILs TLR2, TLR4, and TLR9 and the neonatal neuroimaging findings in cCMV. To the best of our knowledge, this is the first report that links neuroimaging abnormalities and the SNPs of selected cytokines in cCMV. These findings are important to broaden our knowledge of pathogenesis of CNS involvement during cCMV.

Prenatal and neonatal neuroimaging is crucial for the appropriate assessment of a wide range of CNS involvement in cases of cCMV. The most available and safe neuroimaging tool is US, which can detect common abnormalities of cCMV, including cystic lesions, ventriculomegaly, calcifications, and lenticulostriate vasculopathy [42]. Among the study population, 72 infants (78.26%) had abnormal cUS results. We found cystic lesions to be the most common finding on cUS, with over 54% of cases, and ventricular dilatation was the third-most common, with over 30% of cases. Similarly, Smilijkovic et al. noted cystic lesions as the most common US finding, though with a lower frequency (31%) [43]. On the other hand, MRI can add extra information to US, especially when white matter abnormalities and cortical migration defects or cerebral hypoplasia are concerned [3]. Therefore, MRI is utilized to broaden the detection of CNS changes seen in cCMV. It should be underlined that 47% of our patients with normal US had abnormal MRI results. Moreover, Capretti et al. documented that MRI revealed pathological findings in newborns with normal US, with white matter abnormalities being the most frequent [44]. In our study, abnormal white matter was observed in 73.56% of cases, also constituting the most common pathology on MRI, while ventricular dilatation was observed in almost half of the symptomatic patients, being the second-most frequent abnormality. However, Kwak et al. reported a higher rate of ventricular dilatation (67.7%), being the most common abnormal finding on MRI in infants with cCMV [9].

The result of neuroimaging has a crucial impact on therapeutic decisions because one of the important indications for antiviral treatment is evidence of CNS involvement, including radiographic abnormalities consistent with cytomegalovirus CNS disease (ventriculomegaly, intracerebral calcifications, periventricular echogenicity, cortical, or cerebellar malformations). Other signs of CNS disease, such as microcephaly, abnormal cerebrospinal fluid indices for age, chorioretinitis, sensorineural hearing loss, and the detection of cytomegalovirus DNA in cerebrospinal, are also indicative. Recommended treatment duration is 6 months [45,46].

As clinicians taking care of infants with cCMV, we are focused on clinical findings and its unfavorable neurological sequelae, but we also keep in mind the fact that abnormal neuroimaging results are just a consequence of a wide range of factors responsible for brain damaged during cCMV. It has been documented that maternal serological status before pregnancy and the timing of maternal HCMV infection during pregnancy (the earlier the infection, the greater the damage of fetal brain) have an impact [47]. Additionally, HCMV may cause placental damage and dysfunction, including impaired villous growth and differentiation, as well as placental cells apoptosis directly through the viral cytopathic effect or via excessive immune defense [48]. The placental insufficiency has a negative impact on fetal growth and may be a cause of prematurity during maternal HCMV infection [48].

The density of CMV-infected cells and the tropism of HCMV for progenitor cells were the major factors that determined the severity of brain abnormalities, including microcephaly and damage to the olfactory bulbs and the limbic system bulbs at the early period of brain development [49]. Gabrielli et al. reported different varieties and degrees of brain damage in the fetuses with CMV-positive brain tissue [4]. Brain damage correlated with viral tissue load (density of HCMV-positive cells), suggesting a relationship between the severity of infection and histological lesions [4,50]. A very high fetal viral load (CMV-DNA > 50,000 copies/mL) has been observed to occur three times more often in severely damaged fetuses sampled ≤ 28 weeks, but not during the later stages of pregnancy [51]. This means that fetal blood viral load is less predictive of brain damage if sampled in the third trimester. Assessment of viral load in blood of the fetuses with cCMV infection is not a routine procedure in clinical practice, although repeated assessment of viral load in the blood of infants with confirmed cCMV is recommended [45,46]. It should be underlined that a higher viral load might be connected with an increased risk of unfavorable sequelae of cCMV infection [52,53]. The neonatal viral load (>100,000 copies/mL) has been reported as a very good predictor of symptomatic cCMV infection [54]. Moreover, an association between viral load and the number of symptoms has also been reported. The viral load was higher in infants with more presented symptoms [55]. Additionally, a higher viral load has been noted in infants with the presence of CNS involvement during the neonatal period, however no correlation was found between the neonatal viral load and the long-term mental and motor developmental delay [55]. It might also be the case that specific SNPs in cytokines and TLRs affect antiviral defense mechanisms, at least during early life, and have an impact on the viral load and the degree of CNS damage. There is only limited data on the associations between the SNPs of TLRs and the viral load in cCMV infection. The association between the heterozygous variant of TLR9(rs187084) and a higher viral load has been noted in infants with HCMV infection [17]. Furthermore, a higher viral load in infants with the heterozygous genotype of TLR3 (rs377529) and TLR7(rs5741880) has been found [56]. It should be underlined that, not only children with cCMV infection, but also those with postnatal HCMV infection were enrolled into both above-mentioned studies. Interestingly, although G/A variants in TLR2 2258 and TLR9 2848 C > T (rs352140) SNPs correlated with a higher viral load in fetal amniotic fluid, there was no association between TLR2 2258G > A SNP (rs5743708) and the viral load in offspring at birth [20]. In our earlier study [57], we found no associations between neonatal viral load and eight SNPs (included in the current study: IL1B rs16944, IL12B rs3212227, IL28B rs12979860, CCL2 rs1024611, DC-SIGN rs735240, TLR2 rs5743708, TLR4 rs4986791, and TLR9 rs352140). Therefore, the likelihood of the viral load being a confounding factor of neonatal neuroimaging findings seems less probable. Additionally, the repeated regression analysis adjusted for the viral load (data not shown) did not change the outcome of the current study, namely presenting the associations between the SNPs of IL28B and IL1B and the neonatal neuroimaging findings in cCMV.

The CNS involvement during cCMV is multifactorial, but the coexistence of high HCMV densities and high immune cells densities in severely affected brains raises the question of the role of these immune cells in the control of viral replication [58]. The severe cerebral damage with white matter necrosis, polymicrogyria, and microglial nodules included cytomegalic cells surrounded by activated CD8+T-lymphocytes and apoptotic cells, suggesting an immune-mediated injury [4]. CD8+ cells and macrophages are diffused throughout the whole cerebral parenchyma in the most severely affected brains, whereas HCMV-positive cells are preferentially located in the periventricular and germinative areas, irrespective of the severity of the cerebral damage [50]. Human glial and astroglial cells, derived from fetal brain tissue, reply to CMV infection by expressing a number of chemokines and cytokines [1,6]. The predominantly produced chemokine, important in immune cells differentiation and migration, is CCL2 [1,26]; however, we found no association between the SNP of CCL2 and any abnormal neuroimaging findings in the cohort of newborn infants with cCMV. On the other hand, we previously reported an association between CCL2 rs1024611 and sensorineural hearing loss in cCMV [22].

One of the most widely studied proinflammatory cytokines in the brain is IL1. Experimental data suggests that the IL1 family plays a crucial role in MCMV (murine CMV infection) pathogenesis and in the reactivation of a latent virus in adult mice [57], and that IL1 signaling in the CNS elicits responses which can either exacerbate or inhibit neuronal cell apoptosis in neonatal and adult immuno-competent mice [59]. Viral infection is connected with a vigorous inflammatory response such as cellular infiltration and the release of proinflammatory IL1 [60]. A high IL1β level was suggested to provoke the activation of Th1 (T-helper) cells and an inflammatory response [61]. We previously reported that the rare T/T genotype of IL1B rs16944 polymorphism was related to an increased risk of cCMV infection [22]. Additionally, Wujcicka et al. found that the heterozygous C/T genotype of IL1A rs1800587 polymorphism increased the risk of cCMV infection and development of symptomatic infection [25]. By contrast, we recently documented that IL1B rs16944 polymorphism was associated with the reduced risk of splenomegaly [33], suggesting that it has a protective role against the activation of the reticuloendothelial system in cCMV. In the current study, we noted that the SNP polymorphism of IL1B rs16944 was associated with the reduced risk of ventriculomegaly, proposing a protective role against CNS damage in cCMV. Interestingly, all these findings cumulatively suggest that IL1B rs16944 polymorphism may promote cCMV infection [22], while simultaneously reducing the risk of splenomegaly [33] and CNS damage. On the other hand, we found that infants carrying the heterozygous C/T vs. C/C-T/T genotypes in an over-dominant model of IL28B rs12979860 had an increased risk of ventricular dilatation on MRI. Of note is our previous finding that neonates carrying the same heterozygous C/T genotype of IL28B rs12979860 polymorphism demonstrates a greater frequency of thrombocytopenia in comparison to C/C-T/T genotypes [33]. It may indicate that heterozygous IL28B rs12979860 SNP polymorphism is an important factor of the symptomatic course of cCMV, including both CNS and reticuloendothelial system involvement. Interestingly, a homozygous C/C genotype of IL28B rs12979860 was documented as a predictor of viral elimination in patients with hepatitis C virus (HCV) infection [62]. We should consider comprehensive antiviral and cytotoxic actions of IL28 [63] and remember that cytokines induce T-cells that cause chronic inflammation-related tissue damage if unregulated [64]. However, a direct viral toxicity should also be considered, because HCMV-positive cells are preferentially located in the periventricular and germinative areas of fetal brains [50]. Still, additional studies are needed to confirm the relationship between IL28 polymorphisms and abnormal neuroimaging, including periventricular white matter damage which causes ventriculomegaly in cCMV.

As far as other tested SNPs are concerned, we would like to highlight TLRs and their role in cCMV, although we found no association between the SNPs of TLR2, TLR4 or TLR9 and the abnormal neuroimaging results. TLRs play a crucial role in modulating the innate recognition of viruses, not only by serving as pathogen sensors but also by activating signaling pathways that result in an increased production of proinflammatory cytokines and type I IFNs [65]. HCMV triggers cytokine production through the stimulation of pattern recognition receptors (PRRs), most notably Toll-like receptor 2 [64]. The specific SNPs at the TLR-2 genes were associated with the intrauterine transmission of CMV in Israeli pregnant women with primary CMV infection, but only when the onset of infection was limited to the second trimester of pregnancy [37]. Additionally, Wujcicka et al. [20] reported a correlation between the G/A heterozygotic status at TLR2 s5743708 with cCMV infection in Polish fetuses and newborns. The G/A heterozygotic status was eight times more frequent in the infected than in the uninfected patients, and increased the risk of cCMV by ten times. Similarly, the A allele of TLR2 rs5743708 was more than seven times more frequent among the infected cases than in the uninfected ones. Complex AA variants for both TLR2 rs5743708 and TLR9 rs352140 polymorphisms increased the risk of congenital HCMV by more than eleven times. Taking into account the genetic modifications within the TLR2 gene, the CC genotype at SNP rs3804100 in the TLR2 gene was significantly associated with cCMV infection, but not with cCMV disease in a Japanese population. Furthermore, the A/G genotype at SNP rs1898830 in the TLR2 gene tended to be identified less frequently in Japanese children with cCMV infection [32]. The SNP at TLR2 2258 (G/A) might be an important factor associated with an increased risk of cCMV in Polish fetuses and neonates. [20]. Recently, Jabłońska et al. [65] provided the first evidence that SNPs at TLR seem to influence the outcome of the infectious mononucleosis in children and adolescents. Individuals with SNP at TLR4 896 A/G (rs4986790) and TLR9 1174 G/A (rs352139) had a higher risk of the infectious mononucleosis and clinical outcomes when compared with subjects with the wild-type genotype [65]. We found no association between the examined SNPs at the TLR4 and TLR9 genes and the neuroimaging results, although we previously documented that infants carrying the heterozygous C/T genotype at TLR4 rs4986791 had significantly increased odds of CMV-DNA detection in cerebrospinal fluid by more than seven times [66]. Interestingly, when using a multivariate logistic regression analysis, cystic lesions and calcifications on MRI were independent predictors of the positive CMV-PCR results in cerebrospinal fluid [66]. It might be speculated that SNPs of TLR4 have an impact of prolonged presence of CMV-DNA in cerebrospinal fluid and CNS damage in cCMV. An influence of ethnic origin should not be neglected, at least because no statistically significant association between the SNPs at TLR4 and TLR9 genes and cCMV infection or disease was found in a Japanese population [32].

In assessing these study results, it is important to consider the limitations and strengths of the study. Genes examined in this study had been previously verified and some of them had been replicated poorly. Additionally, it might be considered that different host genes might be responsible for the different symptoms of the disease. Moreover, there are many other external factors that could influence the results, such as: HCMV strain virulence, primary or secondary maternal infection, and the trimester of the beginning of cCMV. The study was not designed to address these issues. As far as genetic factors are concerned, the study sample size could not have been powered to detect associations with minor allele genotypes in some genes analyzed that were absent (AA and TT genotypes of TLR2 and TLR4, respectively) or infrequently represented (GG genotype of CCL2) in our study population. Nevertheless, future investigations on neuroimaging findings in cCMV are desired to replicate the abovementioned findings in larger cohorts. Finally, it should be noted that this data set was not adjusted for multiple testing, and so type 1 error cannot be completely ruled out.

The strength of this study is its prospective design with a homogenous population of Caucasian newborn infants with cCMV. We focused only on congenital infection to exclude the influence of probable genetic differences in host response during congenital or acquired HCMV infection. Finally, to the best of our knowledge, this is the first report that links the SNP of selected IL and TLRs with abnormal neuroimaging in cCMV.

## 5. Conclusions

The results of our study demonstrated two novel associations between the SNPs of IL28B and IlB1 and the neonatal neuroimaging results in cCMV: IL1B polymorphism was associated with the reduced risk of ventriculomegaly, while IL28B polymorphism significantly increased the risk of ventriculomegaly on MRI and cystic lesions on US and MRI. Ventricular dilatation on MRI was observed in almost half of the cases, being the second-most common defect after white matter echogenicity on MRI. Conversely, cystic lesions were the most common finding in US. Undoubtedly, further investigations are needed to confirm the described findings in a larger population. From a clinical point of view, additional research on the role of SNPs in the pathogenesis of CNS damage in cCMV infection, including long-term neurological impairment in children, would be beneficial.

## Figures and Tables

**Table 1:** The European MAF values for the SNPs analysed in the study.

Gene dbSNP ID Number	Alleles	MAF *
IL1B rs16944	G/A	A = 0.335
IL12B rs3212227	T/G	G = 0.202
IL28B rs12979860	C/T	T = 0.309
CCL2 rs1024611	A/G	G = 0.271
DC-SIGN rs735240	G/A	A = 0.440
TLR2 rs5743708	G/A	A = 0.029
TLR4 rs4986791	C/T	T = 0.061
TLR9 rs352140	C/T	T = 0.534

MAF, minor allele frequency. * Available from NCBI ALFA project (Release Version: 20201027095038; https://www.ncbi.nlm.nih.gov/snp/docs/gsr/alfa/ (accessed on 16 August 2021)).

**Table 2:** Clinical symptoms and laboratory abnormalities in the study population of newborn infants with congenital HCMV infection.

Symptoms	Study Population (*n* = 92)
microcephaly, n (%)	22 (23.91)
opisthotonos, n (%)	11 (12.09)
abnormal muscle tone, n (%)	53 (58.89)
seizures, n (%)	2 (2.17)
lumbar puncture, n(%)	83 (90.22)
positive PCR (DNA HCMV) in CSF, n (%)	15 (16.3%)
protein level in CSF (>115 mg/dL), n(%)	5 (6.02)
chorioretinitis, n (%)	
at least one eye	17 (18.48)
both eyes	8 (8.70)
abnormal OAE, n (%)	
at least one ear	36 (39.13)
both ears	22 (23.91)
abnormal ABR, n (%)	
at least one ear	29 (34.94) (*n* = 83)
both ears	13 (16.05) (*n* = 81)
IUGR, n (%)	26 (28.26)
petechiae, n (%)	15 (16.30)
thrombocytopenia, n (%)	28 (30.43)
neutropenia, n (%)	10 (10.87)
hepatosplenomegaly, n (%)	17 (18.48)
hepatitis, n (%)	8 (8.79)
cholestasis, n (%)	15 (17.44)
qPCR blood, (×10^3^ copies/mL)	11.5 (2.15–110)
qPCR urine, (×10^6^ copies/mL)	9.0 (1.2–10)

Data are presented as number (%) or median and interquartile range (IQR): CSF, cerebrospinal fluid; OAE, Otoacoustic emission; ABR, Auditory Brainstem Response—threshold for abnormal results > 20dB; thrombocytopenia (<100,000 platelets/mL); neutropenia (<1000 neutrophils/µL); cholestasis (direct bilirubin level > 1.0 mg/dL); hepatitis (elevated level of aspartate transaminase (ASPAT) >84 U/L and/or elevated level of alanine aminotransferase (ALTAT) > 60 U/L); qPCR, quantitative real-time PCR DNA HCMV.

**Table 3:** Neuroimaging findings in newborn infants with cCMV.

Neuroimaging Findings	Study Population (*n* = 92)
abnormal cUS, n (%)	72 (78.26)
calcification, n (%)	25 (27.17)
ventricular dilatation, n (%)	29 (31.52)
cystic lesions, n (%)	50 (54.35)
lenticulostriate vasculopathy, n (%)	41 (44.57)
periventricular echogenicity, n (%)	24 (26.09)
abnormal MRI, n (%)	75 (86.21) (*n* = 87)
calcification, n (%)	15 (17.24)
ventricular dilatation, n (%)	43 (49.43)
cystic lesions, n (%)	26 (29.89)
abnormal white matter, n (%)	64 (73.56)
abnormal myelination, n (%)	15 (17.24)
cortical migration defect, n (%)	12 (13.79)

Data are presented as number (%): cCMV, congenital HCMV infection; cUS, cranial ultrasound; MRI, Magnetic Resonance Imaging.

**Table 4:** Frequencies of genotypes of candidate SNPs in the study population of 92 infants with cCMV.

Gene	dbSNPID Number ^a^	Genotype	n (%)	HWE *p*-Value ^b^
IL1B	rs16944	G/G	36 (39.1%)	0.11
		G/A	49 (53.3%)	
		A/A	7 (7.6%)	
IL12B	rs3212227	T/T	57 (62.0%)	0.23
		T/G	28 (30.4%)	
		G/G	7 (7.6%)	
IL28B	rs12979860	C/C	41 (44.6%)	0.37
		C/T	38 (41.3%)	
		T/T	13 (14.1%)	
CCL2	rs1024611	A/A	50 (54.3%)	0.26
		A/G	39 (42.4%)	
		G/G	3 (3.3%)	
DC-SIGN	rs735240	G/G	35 (38.0%)	0.092
		G/A	37 (40.0%)	
		A/A	20 (22.0%)	
TLR2	rs5743708	G/G	82 (89.1%)	1.0
		G/A	10 (10.9%)	
TLR4	rs4986791	C/C	83 (90.2%)	1.0
		C/T	9 (9.8%)	
TLR9	rs352140	T/T	30 (32.6%)	0.83
		C/T	47 (50.1%)	
		C/C	15 (16.3%)	

cCMV, congenital HCMV infection; HWE, Hardy–Weinberg equilibrium; IL, Interleukin; CCL 2, C-C motif chemokine ligand 2; DC-SIGN, dendritic cell-specific ICAM-grabbing non-integrin; TLR, Toll-like receptor; cUS, cranial ultrasound; ^a^ SNP database (dbSNP) reference number (ID number); ^b^ *p*-value for HWE (*p*-value < 0.05 are considered as significant).

**Table 5:** Associations between SNPs and cystic lesions on cUS in infants with cCMV.

Gene	dbSNPID Number ^a^	Genetic Model	Genotype	Without Cystic Lesins n = 42	Cystic Lesions n = 50	OR (95% CI)	*p*-Value ^b^	AIC
IL1B	rs16944 (G/A)	Codominant	G/G	18 (42.9%)	18 (36%)	1.00	0.56	131.7
			G/A	22 (52.4%)	27 (54%)	1.23 (0.52–2.91)		
			A/A	2 (4.8%)	5 (10%)	2.50 (0.43–14.61)		
		Dominant	G/G	18 (42.9%)	18 (36%)	1.00	0.5	130.4
			G/A-A/A	24 (57.1%)	32 (64%)	1.33 (0.58–3.09)		
		Recessive	G/G-G/A	40 (95.2%)	45 (90%)	1.00	0.34	129.9
			A/A	2 (4.8%)	5 (10%)	2.22 (0.41–12.09)		
		Overdominant	G/G-A/A	20 (47.6%)	23 (46%)	1.00	0.88	130.8
			G/A	22 (52.4%)	27 (54%)	1.07 (0.47–2.43)		
		Log-additive	---	---	---	1.39 (0.70–2.77)	0.34	129.9
IL12B	rs3212227 (T/G)	Codominant	T/T	27 (64.3%)	30 (60%)	1.00	0.64	131.9
			T/G	11 (26.2%)	17 (34%)	1.39 (0.55–3.49)		
			G/G	4 (9.5%)	3 (6%)	0.67 (0.14–3.29)		
		Dominant	T/T	27 (64.3%)	30 (60%)	1.00	0.67	130.7
			T/G-G/G	15 (35.7%)	20 (40%)	1.20 (0.51–2.80)		
		Recessive	T/T-T/G	38 (90.5%)	47 (94%)	1.00	0.53	130.4
			G/G	4 (9.5%)	3 (6%)	0.61 (0.13–2.88)		
		Overdominant	T/T-G/G	31 (73.8%)	33 (66%)	1.00	0.42	130.2
			T/G	11 (26.2%)	17 (34%)	1.45 (0.59–3.58)		
		Log-additive	---	---	---	1.02 (0.53–1.95)	0.95	130.8
IL28B	rs12979860 (C/T)	Codominant	C/C	22 (52.4%)	19 (38%)	1.00	0.015	124.4
			C/T	11 (26.2%)	27 (54%)	2.84 (1.12–7.22)		
			T/T	9 (21.4%)	4 (8%)	0.51 (0.14–1.94)		
		Dominant	C/C	22 (52.4%)	19 (38%)	1.00	0.17	128.9
			C/T-T/T	20 (47.6%)	31 (62%)	1.79 (0.78–4.13)		
		Recessive	C/C-C/T	33 (78.6%)	46 (92%)	1.00	0.064	127.4
			T/T	9 (21.4%)	4 (8%)	0.32 (0.09–1.12)		
		Overdominant	C/C-T/T	31 (73.8%)	23 (46%)	1.00	**0.0064 ^c^**	123.4
			C/T	11 (26.2%)	27 (54%)	3.31 (1.37–8.01)		
		Log-additive	---	---	---	1.02 (0.57–1.83)	0.95	130.8
CCL2	rs1024611 (A/G)	Codominant	A/A	22 (52.4%)	28 (56%)	1.00	0.12	128.6
			G/A	20 (47.6%)	19 (38%)	0.75 (0.32–1.73)		
			G/G	0 (0%)	3 (6%)	NA (0.00–NA)		
		Dominant	A/A	22 (52.4%)	28 (56%)	1.00	0.73	130.7
			A/G-G/G	20 (47.6%)	22 (44%)	0.86 (0.38–1.97)		
		Recessive	A/A-A/G	42 (100%)	47 (94%)	1.00	0.053	127.1
			G/G	0 (0%)	3 (6%)	NA (0.00–NA)		
		Overdominant	A/A-G/G	22 (52.4%)	31 (62%)	1.00	0.35	130
			A/G	20 (47.6%)	19 (38%)	0.67 (0.29–1.55)		
		Log-additive	---	---	---	1.08 (0.52–2.24)	0.84	130.8
DC-SIGN	rs735240 (G/A)	Codominant	G/G	13 (30.9%)	22 (44%)	1.00	0.43	131.2
			G/A	19 (45.2%)	18 (36%)	0.56 (0.22–1.43)		
			A/A	10 (23.8%)	10 (20%)	0.59 (0.19–1.80)		
		Dominant	G/G	13 (30.9%)	22 (44%)	1.00	0.2	129.2
			G/A-A/A	29 (69%)	28 (56%)	0.57 (0.24–1.35)		
		Recessive	G/G-G/A	32 (76.2%)	40 (80%)	1.00	0.66	130.6
			A/A	10 (23.8%)	10 (20%)	0.80 (0.30–2.16)		
		Overdominant	G/G-A/A	23 (54.8%)	32 (64%)	1.00	0.37	130
			G/A	19 (45.2%)	18 (36%)	0.68 (0.29–1.57)		
		Log-additive	---	---	---	0.74 (0.43–1.29)	0.29	129.7
TLR2	rs5743708 (G/A)	---	G/G	38 (90.5%)	44 (88%)	1.00	0.7	130.7
			G/A	4 (9.5%)	6 (12%)	1.30 (0.34–4.94)		
TLR4	rs4986791 (C/T)	---	C/C	37 (88.1%)	46 (92%)	1.00	0.53	130.4
			T/C	5 (11.9%)	4 (8%)	0.64 (0.16–2.57)		
TLR9	rs352140 (C/T)	Codominant	T/T	10 (23.8%)	20 (40%)	1.00	0.23	129.9
			C/T	25 (59.5%)	22 (44%)	0.44 (0.17–1.14)		
			C/C	7 (16.7%)	8 (16%)	0.57 (0.16–2.03)		
		Dominant	T/T	10 (23.8%)	20 (40%)	1.00	0.096	128.1
			C/T-C/C	32 (76.2%)	30 (60%)	0.47 (0.19–1.16)		
		Recessive	T/T-C/T	35 (83.3%)	42 (84%)	1.00	0.93	130.8
			C/C	7 (16.7%)	8 (16%)	0.95 (0.31–2.89)		
		Overdominant	T/T-C/C	17 (40.5%)	28 (56%)	1.00	0.14	128.6
			C/T	25 (59.5%)	22 (44%)	0.53 (0.23–1.23)		
		Log-additive	---	---	---	0.69 (0.38–1.28)	0.24	129.4

Data presented as number (%): cUS, cranial ultrasound; OR, odds ratio; CI, confidence interval; AIC, Akaike information criteria; NA, not applicable; IL, Interleukin; CCL 2, C-C motif chemokine ligand 2; DC-SIGN, dendritic cell-specific ICAM-grabbing non-integrin; TLR, Toll-like receptor; cUS, cranial ultrasound; ^a^ SNP database (dbSNP) reference number (ID number); ^b^ P-value for comparison between infants with and without cystic lesions; ^c^ The best fitted model based on AIC. Bold in table represents statistically significant.

**Table 6:** Associations between SNPs and ventricular dilatation on cUS in infants with cCMV.

Gene	dbSNPID Number ^a^	Genetic Model	Genotype	Without Ventricular Dilatation n = 63	Ventricular Dilatation n = 29	OR (95% CI)	*p*-Value ^b^	AIC
IL1B	rs16944 (G/A)	Codominant	G/G	20 (31.8%)	16 (55.2%)	1.00	0.098	116
			G/A	38 (60.3%)	11 (37.9%)	0.36 (0.14–0.93)		
			A/A	5 (7.9%)	2 (6.9%)	0.50 (0.09–2.93)		
		Dominant	G/G	20 (31.8%)	16 (55.2%)	1.00	**0.034 ^c^**	114.1
			G/A-A/A	43 (68.2%)	13 (44.8%)	0.38 (0.15–0.93)		
		Recessive	G/G-G/A	58 (92.1%)	27 (93.1%)	1.00	0.86	118.6
			A/A	5 (7.9%)	2 (6.9%)	0.86 (0.16–4.71)		
		Overdominant	G/G-A/A	25 (39.7%)	18 (62.1%)	1.00	0.045	114.7
			G/A	38 (60.3%)	11 (37.9%)	0.40 (0.16–0.99)		
		Log-additive	---	---	---	0.50 (0.23–1.08)	0.068	115.3
IL12B	rs3212227 (T/G)	Codominant	T/T	37 (58.7%)	20 (69%)	1.00	0.46	119.1
			T/G	20 (31.8%)	8 (27.6%)	0.74 (0.28–1.98)		
			G/G	6 (9.5%)	1 (3.5%)	0.31 (0.03–2.74)		
		Dominant	T/T	37 (58.7%)	20 (69%)	1.00	0.34	117.8
			T/G-G/G	26 (41.3%)	9 (31%)	0.64 (0.25–1.63)		
		Recessive	T/T-T/G	57 (90.5%)	28 (96.5%)	1.00	0.28	117.5
			G/G	6 (9.5%)	1 (3.5%)	0.34 (0.04–2.96)		
		Overdominant	T/T-G/G	43 (68.2%)	21 (72.4%)	1.00	0.69	118.5
			T/G	20 (31.8%)	8 (27.6%)	0.82 (0.31–2.16)		
		Log-additive	---	---	---	0.65 (0.30–1.37)	0.24	117.3
IL28B	rs12979860 (C/T)	Codominant	C/C	32 (50.8%)	9 (31%)	1.00	0.17	117.1
			C/T	24 (38.1%)	14 (48.3%)	2.07 (0.77–5.59)		
			T/T	7 (11.1%)	6 (20.7%)	3.05 (0.82–11.38)		
		Dominant	C/C	32 (50.8%)	9 (31%)	1.00	0.073	115.5
			C/T-T/T	31 (49.2%)	20 (69%)	2.29 (0.91–5.81)		
		Recessive	C/C-C/T	56 (88.9%)	23 (79.3%)	1.00	0.23	117.2
			T/T	7 (11.1%)	6 (20.7%)	2.09 (0.63–6.88)		
		Overdominant	C/C-T/T	39 (61.9%)	15 (51.7%)	1.00	0.36	117.8
			C/T	24 (38.1%)	14 (48.3%)	1.52 (0.62–3.69)		
		Log-additive	---	---	---	1.80 (0.96–3.30)	0.064	115.2
CCL2	rs1024611 (A/G)	Codominant	A/A	37 (58.7%)	13 (44.8%)	1.00	0.1	116.1
			A/G	23 (36.5%)	16 (55.2%)	1.98 (0.81–4.86)		
			G/G	3 (4.8%)	0 (0%)	0.00 (0.00-NA)		
		Dominant	A/A	37 (58.7%)	13 (44.8%)	1.00	0.21	117.1
			A/G-G/G	26 (41.3%)	16 (55.2%)	1.75 (0.72–4.25)		
		Recessive	A/A-A/G	60 (95.2%)	29 (100%)	1.00	0.13	116.4
			G/G	3 (4.8%)	0 (0%)	0.00 (0.00-NA)		
		Overdominant	A/A-G/G	40 (63.5%)	13 (44.8%)	1.00	0.093	115.9
			A/G	23 (36.5%)	16 (55.2%)	2.14 (0.88–5.23)		
		Log-additive	---	---	---	1.33 (0.61–2.89)	0.47	118.1
DC-SIGN	rs735240 (G/A)	Codominant	G/G	28 (44.4%)	7 (24.1%)	1.00	0.16	117
			G/A	23 (36.5%)	14 (48.3%)	2.43 (0.84–7.04)		
			A/A	12 (19.1%)	8 (27.6%)	2.67 (0.79–9.02)		
		Dominant	G/G	28 (44.4%)	7 (24.1%)	1.00	0.057	115.1
			G/A-A/A	35 (55.6%)	22 (75.9%)	2.51 (0.94–6.73)		
		Recessive	G/G-G/A	51 (81%)	21 (72.4%)	1.00	0.36	117.8
			A/A	12 (19.1%)	8 (27.6%)	1.62 (0.58–4.53)		
		Overdominant	G/G-A/A	40 (63.5%)	15 (51.7%)	1.00	0.29	117.5
			G/A	23 (36.5%)	14 (48.3%)	1.62 (0.67–3.96)		
		Log-additive	---	---	---	1.66 (0.92–3.00)	0.089	115.8
TLR2	rs5743708 (G/A)	---	G/G	57 (90.5%)	25 (86.2%)	1.00	0.55	118.3
			G/A	6 (9.5%)	4 (13.8%)	1.52 (0.39–5.86)		
TLR4	rs4986791 (C/T)	---	C/C	58 (92.1%)	25 (86.2%)	1.00	0.39	117.9
			C/T	5 (7.9%)	4 (13.8%)	1.86 (0.46–7.50)		
TLR9	rs352140 (C/T)	Codominant	T/T	19 (30.2%)	11 (37.9%)	1.00	0.2	117.5
			C/T	31 (49.2%)	16 (55.2%)	0.89 (0.34–2.32)		
			C/C	13 (20.6%)	2 (6.9%)	0.27 (0.05–1.40)		
		Dominant	T/T	19 (30.2%)	11 (37.9%)	1.00	0.46	118.1
			C/T-C/C	44 (69.8%)	18 (62.1%)	0.71 (0.28–1.78)		
		Recessive	T/T-C/T	50 (79.4%)	27 (93.1%)	1.00	0.077	115.5
			C/C	13 (20.6%)	2 (6.9%)	0.28 (0.06–1.36)		
		Overdominant	T/T-C/C	32 (50.8%)	13 (44.8%)	1.00	0.59	118.4
			C/T	31 (49.2%)	16 (55.2%)	1.27 (0.53–3.07)		
		Log-additive	---	---	---	0.62 (0.32–1.21)	0.16	116.6

Data presented as number (%): cUS, cranial ultrasound; cCMV, congenital HCMV infection; OR, odds ratio; CI, confidence interval; NA, not applicable; AIC, Akaike information criteria; IL, Interleukin; CCL 2, C-C motif chemokine ligand 2; DC-SIGN, dendritic cell-specific ICAM-grabbing non-integrin; TLR, Toll-like receptor; ^a^ SNP database (dbSNP) reference number (ID number); ^b^ *p*-value for comparison between asymptomatic and symptomatic group; ^c^ The best fitted model based on AIC. Bold in table represents statistically significant.

**Table 7:** Associations between SNPs and ventricular dilatation on MRI in infants with cCMV.

Gene	dbSNPID Number ^a^	Genetic Model	Genotype	Without Ventricular Dilatation n = 44	Ventricular Dilatation n = 43	OR (95% CI)	*p*-Value ^b^	AIC
IL1B	rs16944 (G/A)	Codominant	G/G	13 (29.6%)	22 (51.2%)	1.00	0.093	121.8
			G/A	26 (59.1%)	19 (44.2%)	0.43 (0.17–1.07)		
			A/A	5 (11.4%)	2 (4.7%)	0.24 (0.04–1.40)		
		Dominant	G/G	13 (29.6%)	22 (51.2%)	1.00	0.039	120.3
			G/A-A/A	31 (70.5%)	21 (48.8%)	0.40 (0.17–0.97)		
		Recessive	G/G-G/A	39 (88.6%)	41 (95.3%)	1.00	0.24	123.2
			A/A	5 (11.4%)	2 (4.7%)	0.38 (0.07–2.08)		
		Overdominant	G/G-A/A	18 (40.9%)	24 (55.8%)	1.00	0.16	122.7
			G/A	26 (59.1%)	19 (44.2%)	0.55 (0.23–1.28)		
		Log-additive	---	---	---	0.46 (0.22–0.95)	**0.03 ^c^**	119.9
IL12B	rs3212227 (T/G)	Codominant	T/T	27 (61.4%)	26 (60.5%)	1.00	0.43	124.9
			T/G	12 (27.3%)	15 (34.9%)	1.30 (0.51–3.29)		
			G/G	5 (11.4%)	2 (4.7%)	0.42 (0.07–2.33)		
		Dominant	T/T	27 (61.4%)	26 (60.5%)	1.00	0.93	124.6
			T/G-G/G	17 (38.6%)	17 (39.5%)	1.04 (0.44–2.46)		
		Recessive	T/T-T/G	39 (88.6%)	41 (95.3%)	1.00	0.24	123.2
			G/G	5 (11.4%)	2 (4.7%)	0.38 (0.07–2.08)		
		Overdominant	T/T-G/G	32 (72.7%)	28 (65.1%)	1.00	0.44	124
			T/G	12 (27.3%)	15 (34.9%)	1.43 (0.57–3.56)		
		Log-additive	---	---	---	0.87 (0.45–1.68)	0.67	124.4
IL28B	rs12979860 (C/T)	Codominant	C/C	23 (52.3%)	14 (32.6%)	1.00	0.11	122.1
			C/T	14 (31.8%)	23 (53.5%)	2.70 (1.05–6.91)		
			T/T	7 (15.9%)	6 (13.9%)	1.41 (0.39–5.05)		
		Dominant	C/C	23 (52.3%)	14 (32.6%)	1.00	0.062	121.1
			C/T-T/T	21 (47.7%)	29 (67.4%)	2.27 (0.95–5.41)		
		Recessive	C/C-C/T	37 (84.1%)	37 (86.1%)	1.00	0.8	124.5
			T/T	7 (15.9%)	6 (13.9%)	0.86 (0.26–2.79)		
		Overdominant	C/C-T/T	30 (68.2%)	20 (46.5%)	1.00	**0.04 ^c^**	120.4
			C/T	14 (31.8%)	23 (53.5%)	2.46 (1.03–5.90)		
		Log-additive	---	---	---	1.43 (0.78–2.62)	0.24	123.2
CCL2	rs1024611 (A/G)	Codominant	A/A	21 (47.7%)	24 (55.8%)	1.00	0.56	125.4
			G/A	22 (50%)	17 (39.5%)	0.68 (0.29–1.60)		
			G/G	1 (2.3%)	2 (4.7%)	1.75 (0.15–20.71)		
		Dominant	A/A	21 (47.7%)	24 (55.8%)	1.00	0.45	124
			A/G-G/G	23 (52.3%)	19 (44.2%)	0.72 (0.31–1.68)		
		Recessive	A/A-A/G	43 (97.7%)	41 (95.3%)	1.00	0.54	124.2
			G/G	1 (2.3%)	2 (4.7%)	2.10 (0.18–24.03)		
		Overdominant	A/A-G/G	22 (50%)	26 (60.5%)	1.00	0.33	123.6
			A/G	22 (50%)	17 (39.5%)	0.65 (0.28–1.53)		
		Log-additive	---	---	---	0.84 (0.40–1.76)	0.64	124.4
DC-SIGN	rs735240 (G/A)	Codominant	G/G	15 (34.1%)	16 (37.2%)	1.00	0.72	125.9
			G/A	20 (45.5%)	16 (37.2%)	0.75 (0.29–1.97)		
			A/A	9 (20.4%)	11 (25.6%)	1.15 (0.37–3.54)		
		Dominant	G/G	15 (34.1%)	16 (37.2%)	1.00	0.76	124.5
			G/A-A/A	29 (65.9%)	27 (62.8%)	0.87 (0.36–2.10)		
		Recessive	G/G-G/A	35 (79.5%)	32 (74.4%)	1.00	0.57	124.3
			A/A	9 (20.4%)	11 (25.6%)	1.34 (0.49–3.64)		
		Overdominant	G/G-A/A	24 (54.5%)	27 (62.8%)	1.00	0.43	124
			G/A	20 (45.5%)	16 (37.2%)	0.71 (0.30–1.68)		
		Log-additive	---	---	---	1.04 (0.59–1.81)	0.9	124.6
TLR2	rs5743708 (G/A)	---	G/G	40 (90.9%)	38 (88.4%)	1.00	0.7	124.4
			G/A	4 (9.1%)	5 (11.6%)	1.32 (0.33–5.27)		
TLR4	rs4986791 (C/T)	---	C/C	42 (95.5%)	37 (86%)	1.00	0.12	122.2
			C/T	2 (4.5%)	6 (13.9%)	3.41 (0.65–17.91)		
TLR9	rs352140 (C/T)	Codominant	T/T	13 (29.6%)	15 (34.9%)	1.00	0.38	124.7
			C/T	21 (47.7%)	23 (53.5%)	0.95 (0.37–2.45)		
			C/C	10 (22.7%)	5 (11.6%)	0.43 (0.12–1.60)		
		Dominant	T/T	13 (29.6%)	15 (34.9%)	1.00	0.59	124.3
			C/T-C/C	31 (70.5%)	28 (65.1%)	0.78 (0.32–1.93)		
		Recessive	T/T-C/T	34 (77.3%)	38 (88.4%)	1.00	0.17	122.7
			C/C	10 (22.7%)	5 (11.6%)	0.45 (0.14–1.44)		
		Overdominant	T/T-C/C	23 (52.3%)	20 (46.5%)	1.00	0.59	124.3
			C/T	21 (47.7%)	23 (53.5%)	1.26 (0.54–2.92)		
		Log-additive	---	---	---	0.70 (0.38–1.31)	0.26	123.3

Data presented as number (%): OR, odds ratio; CI, confidence interval; AIC, Akaike information criteria; NA, not applicable; NS, not significant (*p*-values above 0.05); IL, Interleukin; CCL 2, C-C motif chemokine ligand 2; DC-SIGN, dendritic cell-specific ICAM-grabbing non-integrin; TLR, Toll-like receptor; MRI, brain magnetic resonance imaging; ^a^ SNP database (dbSNP) reference number (ID number); ^b^ *p*-value for comparison between infants with and without ventricular dilatation; *p*-value <0.05 are considered statistically significant; ^c^ The best fitted model based on AIC. Bold in table represents statistically significant.

**Table 8:** Associations between SNPs and cystic lesions on MRI in infants with cCMV.

Gene	dbSNPID Number ^a^	Genetic Model	Genotype	Without Cystic Lesions n = 61	Cystic Lesions n = 26	OR (95% CI)	*p*-Value ^b^	AIC
IL1B	rs16944 (G/A)	Codominant	G/G	24 (39.3%)	11 (42.3%)	1.00	0.97	112.1
			G/A	32 (52.5%)	13 (50%)	0.89 (0.34–2.32)		
			A/A	5 (8.2%)	2 (7.7%)	0.87 (0.15–5.22)		
		Dominant	G/G	24 (39.3%)	11 (42.3%)	1.00	0.8	110.1
			G/A-A/A	37 (60.7%)	15 (57.7%)	0.88 (0.35–2.25)		
		Recessive	G/G-G/A	56 (91.8%)	24 (92.3%)	1.00	0.94	110.1
			A/A	5 (8.2%)	2 (7.7%)	0.93 (0.17–5.15)		
		Overdominant	G/G-A/A	29 (47.5%)	13 (50%)	1.00	0.83	110.1
			G/A	32 (52.5%)	13 (50%)	0.91 (0.36–2.27)		
		Log-additive	---	---	---	0.91 (0.43–1.93)	0.81	110.1
IL12B	rs3212227 (T/G)	Codominant	T/T	34 (55.7%)	19 (73.1%)	1.00	0.26	109.4
			T/G	22 (36.1%)	5 (19.2%)	0.41 (0.13–1.25)		
			G/G	5 (8.2%)	2 (7.7%)	0.72 (0.13–4.05)		
		Dominant	T/T	34 (55.7%)	19 (73.1%)	1.00	0.12	107.7
			T/G-G/G	27 (44.3%)	7 (26.9%)	0.46 (0.17–1.27)		
		Recessive	T/T-T/G	56 (91.8%)	24 (92.3%)	1.00	0.94	110.1
			G/G	5 (8.2%)	2 (7.7%)	0.93 (0.17–5.15)		
		Overdominant	T/T-G/G	39 (63.9%)	21 (80.8%)	1.00	0.11	107.6
			T/G	22 (36.1%)	5 (19.2%)	0.42 (0.14–1.28)		
		Log-additive	---	---	---	0.62 (0.28–1.37)	0.22	108.6
IL28B	rs12979860 (C/T)	Codominant	C/C	31 (50.8%)	6 (23.1%)	1.00	0.0043	101.2
			C/T	19 (31.1%)	18 (69.2%)	4.89 (1.65–14.50)		
			T/T	11 (18%)	2 (7.7%)	0.94 (0.16–5.36)		
		Dominant	C/C	31 (50.8%)	6 (23.1%)	1.00	0.014	104.1
			C/T-T/T	30 (49.2%)	20 (76.9%)	3.44 (1.22–9.76)		
		Recessive	C/C-C/T	50 (82%)	24 (92.3%)	1.00	0.19	108.4
			T/T	11 (18%)	2 (7.7%)	0.38 (0.08–1.85)		
		Overdominant	C/C-T/T	42 (68.8%)	8 (30.8%)	1.00	**0.001 ^c^**	99.2
			C/T	19 (31.1%)	18 (69.2%)	4.97 (1.84–13.43)		
		Log-additive	---	---	---	1.41 (1.84–13.43)	0.3	109.0
CCL2	rs1024611 (A/G)	Codominant	A/A	30 (49.2%)	15 (57.7%)	1.00	0.0098 ^d^	102.9
			A/G	31 (50.8%)	8 (30.8%)	0.52 (0.19–1.39)		
			G/G	0 (0%)	3 (11.5%)	NA (0.00-NA)		
		Dominant	A/A	30 (49.2%)	15 (57.7%)	1.00	0.47	109.6
			A/G-G/G	31 (50.8%)	11 (42.3%)	0.71 (0.28–1.79)		
		Recessive	A/A-A/G	61 (100%)	23 (88.5%)	1.00	0.0062	102.6
			G/G	0 (0%)	3 (11.5%)	NA (0.00-NA)		
		Overdominant	A/A-G/G	30 (49.2%)	18 (69.2%)	1.00	0.082	107.1
			A/G	31 (50.8%)	8 (30.8%)	0.43 (0.16–1.14)		
		Log-additive	---	---	---	1.10 (0.49–2.47)	0.82	110.1
DC-SIGN	rs735240 (G/A)	Codominant	A/A	30 (49.2%)	15 (57.7%)	1.00	0.0098 ^d^	102.9
			G/A	31 (50.8%)	8 (30.8%)	0.52 (0.19–1.39)		
			G/G	0 (0%)	3 (11.5%)	NA (0.00-NA)		
		Dominant	A/A	30 (49.2%)	15 (57.7%)	1.00	0.47	109.6
			G/A-G/G	31 (50.8%)	11 (42.3%)	0.71 (0.28–1.79)		
		Recessive	A/A-G/A	61 (100%)	23 (88.5%)	1.00	0.0062	102.6
			G/G	0 (0%)	3 (11.5%)	NA (0.00-NA)		
		Overdominant	A/A-G/G	30 (49.2%)	18 (69.2%)	1.00	0.082	107.1
			G/A	31 (50.8%)	8 (30.8%)	0.43 (0.16–1.14)		
		Log-additive	---	---	---	1.10 (0.49–2.47)	0.82	110.1
TLR2	rs5743708 (G/A)	---	G/G	55 (90.2%)	23 (88.5%)	1.00	0.81	110.1
			G/A	6 (9.8%)	3 (11.5%)	1.20 (0.28–5.19)		
TLR4	rs4986791 (C/T)	---	C/C	56 (91.8%)	23 (88.5%)	1.00	0.63	109.9
			C/T	5 (8.2%)	3 (11.5%)	1.46 (0.32–6.62)		
TLR9	rs352140 (C/T)	Codominant	T/T	17 (27.9%)	11 (42.3%)	1.00	0.36	110.1
			C/T	32 (52.5%)	12 (46.1%)	0.58 (0.21–1.59)		
			C/C	12 (19.7%)	3 (11.5%)	0.39 (0.09–1.69)		
		Dominant	T/T	17 (27.9%)	11 (42.3%)	1.00	0.19	108.4
			C/T-C/C	44 (72.1%)	15 (57.7%)	0.53 (0.20–1.37)		
		Recessive	T/T-C/T	49 (80.3%)	23 (88.5%)	1.00	0.34	109.2
			C/C	12 (19.7%)	3 (11.5%)	0.53 (0.14–2.07)		
		Overdominant	T/T-C/C	29 (47.5%)	14 (53.9%)	1.00	0.59	109.8
			C/T	32 (52.5%)	12 (46.1%)	0.78 (0.31–1.95)		
		Log-additive	---	---	---	0.61 (0.30–1.22)	0.16	108.1

Data presented as number (%): MRI, magnetic resonance imaging; cCMV, congenital HCMV infection; OR, odds ratio; CI, confidence interval; NA, not applicable; AIC, Akaike information criteria; IL, Interleukin; CCL 2, C-C motif chemokine ligand 2; DC-SIGN, dendritic cell-specific ICAM-grabbing non-integrin; TLR, Toll-like receptor; ^a^ SNP database (dbSNP) reference number (ID number); ^b^ *p*-value for comparison between asymptomatic and symptomatic group; ^c^ The best fitted model based on AIC. ^d^ Since 1 (the value of ‘no effect”) lies between the confidence limits, the association for this SNP is not considered significant. Bold in table represents statistically significant.

## Data Availability

All data from this study is included in the current manuscript.

## Supplementary Materials

The following are available online at https://www.mdpi.com/article/10.3390/v13091783/s1, Table S1: Associations between SNPs and lenticulostriate vasculopathy (LSV) on cUS in infants with cCMV. Table S2: Associations between SNPs and increased periventricular echogenicity on cUS in infants with cCMV. Table S3: Associations between SNPs and calcifications on cUS in infants with cCMV. Table S4: Associations between SNPs and abnormal myelination on MRI in infants with cCMV. Table S5: Associations between SNPs and abnormal white matter on MRI in infants with cCMV. Table S6: Associations between SNPs and calcification on MRI in infants with cCMV. Table S7: Associations between SNPs and cortical migration defect on MRI in infants with cCMV.
