# Supplementary material for: Single Nucleotide Polymorphisms of Interleukins and Toll-like Receptors and Neuroimaging Results in Newborns with Congenital HCMV Infection
Source: Viruses. 2021 Sep 7;13(9):1783. doi: 10.3390/v13091783 (PMC8473223; doi:10.3390/v13091783)
Supplement: Supplementary file 1 [file viruses-13-01783-s001.zip › viruses-1340800-SI.pdf]

Table S1. Associations between SNPs and lenticulostriate vasculopathy (LSV) on cUS in infants with cCMV.

Gene	dbSNP ID number[1]	Genetic Model	Genotype	Without LSV *n* =51	LSV *n* = 41	OR (95% CI)	*P*-value[2]	AIC
IL1B	rs16944 (G/A)	Codominant	G/G	19 (37.2%)	17 (41.5%)	1.00	0.65	131.6
			G/A	27 (52.9%)	22 (53.7%)	0.91 (0.38-2.16)		
			A/A	5 (9.8%)	2 (4.9%)	0.45 (0.08-2.61)		
		Dominant	G/G	19 (37.2%)	17 (41.5%)	1.00	0.68	130.3
			G/A-A/A	32 (62.8%)	24 (58.5%)	0.84 (0.36-1.94)		
		Recessive	G/G-G/A	46 (90.2%)	39 (95.1%)	1.00	0.37	129.6
			A/A	5 (9.8%)	2 (4.9%)	0.47 (0.09-2.57)		
		Overdominant	G/G-A/A	24 (47.1%)	19 (46.3%)	1.00	0.95	130.4
			G/A	27 (52.9%)	22 (53.7%)	1.03 (0.45-2.35)		
		Log-additive	---	---	---	0.78 (0.39-1.54)	0.47	129.9
IL12B	rs3212227 (T/G)	Codominant	T/T	33 (64.7%)	24 (58.5%)	1.00	0.79	132
			T/G	14 (27.4%)	14 (34.1%)	1.37 (0.55-3.41)		
			G/G	4 (7.8%)	3 (7.3%)	1.03 (0.21-5.04)		
		Dominant	T/T	33 (64.7%)	24 (58.5%)	1.00	0.54	130.1
			T/G-G/G	18 (35.3%)	17 (41.5%)	1.30 (0.56-3.03)		
		Recessive	T/T-T/G	47 (92.2%)	38 (92.7%)	1.00	0.92	130.4
			G/G	4 (7.8%)	3 (7.3%)	0.93 (0.20-4.40)		
		Overdominant	T/T-G/G	37 (72.5%)	27 (65.8%)	1.00	0.49	130
			T/G	14 (27.4%)	14 (34.1%)	1.37 (0.56-3.34)		
		Log-additive	---	---	---	1.15 (0.60-2.20)	0.67	130.3
IL28B	rs12979860 (C/T)	Codominant	C/C	25 (49%)	16 (39%)	1.00	0.61	131.5
			C/T	19 (37.2%)	19 (46.3%)	1.56 (0.64-3.82)		
			T/T	7 (13.7%)	6 (14.6%)	1.34 (0.38-4.71)		
		Dominant	C/C	25 (49%)	16 (39%)	1.00	0.34	129.5
			C/T-T/T	26 (51%)	25 (61%)	1.50 (0.65-3.46)		
		Recessive	C/C-C/T	44 (86.3%)	35 (85.4%)	1.00	0.9	130.4
			T/T	7 (13.7%)	6 (14.6%)	1.08 (0.33-3.50)		
		Overdominant	C/C-T/T	32 (62.8%)	22 (53.7%)	1.00	0.38	129.7
		Log-additive	---	---	---	1.25 (0.69-2.24)	0.46	129.9
CCL2	rs1024611 (A/G)	Codominant	A/A	29 (56.9%)	21 (51.2%)	1.00	0.68	131.7
			A/G	21 (41.2%)	18 (43.9%)	1.18 (0.51-2.75)		
			G/G	1 (2%)	2 (4.9%)	2.76 (0.23-32.50)		
		Dominant	A/A	29 (56.9%)	21 (51.2%)	1.00	0.59	130.2
			A/G-G/G	22 (43.1%)	20 (48.8%)	1.26 (0.55-2.87)		
		Recessive	A/A-A/G	50 (98%)	39 (95.1%)	1.00	0.43	129.8
			G/G	1 (2%)	2 (4.9%)	2.56 (0.22-29.32)		
		Overdominant	A/A-G/G	30 (58.8%)	23 (56.1%)	1.00	0.79	130.4
			A/G	21 (41.2%)	18 (43.9%)	1.12 (0.49-2.57)		
		Log-additive	---	---	---	1.31 (0.63-2.73)	0.47	129.9
DC-SIGN	rs735240 (G/A)	Codominant	G/G	19 (37.2%)	16 (39%)	1.00	0.78	131.9
			G/A	22 (43.1%)	15 (36.6%)	0.81 (0.32-2.06)		

			A/A	10 (19.6%)	10 (24.4%)	1.19 (0.40-3.57)		
		Dominant	G/G	19 (37.2%)	16 (39%)	1.00	0.86	130.4
			G/A-A/A	32 (62.8%)	25 (61%)	0.93 (0.40-2.16)		
		Recessive	G/G-G/A	41 (80.4%)	31 (75.6%)	1.00	0.58	130.1
			A/A	10 (19.6%)	10 (24.4%)	1.32 (0.49-3.57)		
		Overdominant	G/G-A/A	29 (56.9%)	26 (63.4%)	1.00	0.52	130
			G/A	22 (43.1%)	15 (36.6%)	0.76 (0.33-1.77)		
		Log-additive	---	---	---	1.05 (0.61-1.82)	0.85	130.4
TLR2	rs5743708 (G/A)	---	G/G	48 (94.1%)	34 (82.9%)	1.00	0.085	127.5
			G/A	3 (5.9%)	7 (17.1%)	3.29 (0.79-13.66)		
TLR4	rs4986791 (C/T)	---	C/C	47 (92.2%)	36 (87.8%)	1.00	0.49	130
			C/T	4 (7.8%)	5 (12.2%)	1.63 (0.41-6.52)		
TLR9	rs352140 (C/T)	Codominant	T/T	17 (33.3%)	13 (31.7%)	1.00	0.98	132.4
			C/T	26 (51%)	21 (51.2%)	1.06 (0.42-2.66)		
			C/C	8 (15.7%)	7 (17.1%)	1.14 (0.33-3.97)		
		Dominant	T/T	17 (33.3%)	13 (31.7%)	1.00	0.87	130.4
			C/T-C/C	34 (66.7%)	28 (68.3%)	1.08 (0.45-2.59)		
		Recessive	T/T-C/T	43 (84.3%)	34 (82.9%)	1.00	0.86	130.4
			C/C	8 (15.7%)	7 (17.1%)	1.11 (0.36-3.36)		
		Overdominant	T/T-C/C	25 (49%)	20 (48.8%)	1.00	0.98	130.4
			C/T	26 (51%)	21 (51.2%)	1.01 (0.44-2.30)		
		Log-additive	---	---	---	1.07 (0.58-1.95)	0.83	130.4

Data presented as number (%), cUS, cranial ultrasound; cCMV, congenital HCMV infection; LSV, lenticulostriate vasculopathy; OR, odds ratio; CI, confidence interval; NA, not applicable; AIC, Akaike information criteria; IL, Interleukin; CCL 2, C-C motif chemokine ligand 2; DC-SIGN, dendritic cell-specific ICAM-grabbing non-integrin; TLR, Toll-like receptor; [1] SNP database (dbSNP) reference number (ID number); [2] P-value for comparison between asymptomatic and symptomatic group;

Table S2. Associations between SNPs and increased periventricular echogenicity on cUS in infants with cCMV.

Gene	dbSNP ID number[1]	Genetic Model	Genotype	Without increased periventricular echogenicity *n* = 68	Increased periventricular echogenicity *n* = 24	OR (95% CI)	*P*-value[2]	AIC
IL1B	rs16944 (G/A)	Codominant	G/G	26 (38.2%)	10 (41.7%)	1.00	0.93	111.5
			G/A	37 (54.4%)	12 (50%)	0.84 (0.32-2.24)		
			A/A	5 (7.3%)	2 (8.3%)	1.04 (0.17-6.26)		
		Dominant	G/G	26 (38.2%)	10 (41.7%)	1.00	0.77	109.5
			G/A-A/A	42 (61.8%)	14 (58.3%)	0.87 (0.34-2.24)		
		Recessive	G/G G/A	63 (92.7%)	22 (91.7%)	1.00	0.88	109.6
			A/A	5 (7.3%)	2 (8.3%)	1.15 (0.21-6.33)		
		Overdominant	G/G-A/A	31 (45.6%)	12 (50%)	1.00	0.71	109.5
			G/A	37 (54.4%)	12 (50%)	0.84 (0.33-2.13)		
		Log-additive	---	---	---	0.94 (0.43-2.02)	0.86	109.6
IL12B	rs3212227 (T/G)	Codominant	T/T	39 (57.4%)	18 (75%)	1.00	0.067	106.2
			T/G	22 (32.4%)	6 (25%)	0.59 (0.20-1.71)		
			G/G	7 (10.3%)	0 (0%)	0.00 (0.00-NA)		
		Dominant	T/T	39 (57.4%)	18 (75%)	1.00	0.12	107.2
			T/G-G/G	29 (42.6%)	6 (25%)	0.45 (0.16-1.27)		

		Recessive	T/T-T/G	61 (89.7%)	24 (100%)	1.00	0.035	105.2
			G/G	7 (10.3%)	0 (0%)	0.00 (0.00-NA)		
		Overdominant	T/T-G/G	46 (67.7%)	18 (75%)	1.00	0.5	109.1
			T/G	22 (32.4%)	6 (25%)	0.70 (0.24-2.00)		
		Log-additive	---	---	---	0.43 (0.17-1.08)	0.049	105.7
IL28B	rs12979860 (C/T)	Codominant	C/C	30 (44.1%)	11 (45.8%)	1.00	0.6	110.6
			C/T	27 (39.7%)	11 (45.8%)	1.11 (0.42-2.97)		
			T/T	11 (16.2%)	2 (8.3%)	0.50 (0.09-2.60)		
		Dominant	C/C	30 (44.1%)	11 (45.8%)	1.00	0.88	109.6
			C/T-T/T	38 (55.9%)	13 (54.2%)	0.93 (0.37-2.38)		
		Recessive	C/C-C/T	57 (83.8%)	22 (91.7%)	1.00	0.32	108.6
			T/T	11 (16.2%)	2 (8.3%)	0.47 (0.10-2.30)		
		Overdominant	C/C-T/T	41 (60.3%)	13 (54.2%)	1.00	0.6	109.3
			C/T	27 (39.7%)	11 (45.8%)	1.28 (0.50-3.28)		
		Log-additive	---	---	---	0.82 (0.42-1.61)	0.56	109.3
CCL2	rs1024611 (A/G)	Codominant	A/A	35 (51.5%)	15 (62.5%)	1.00	0.3	109.2
			A/G	30 (44.1%)	9 (37.5%)	0.70 (0.27-1.83)		
			G/G	3 (4.4%)	0 (0%)	0.00 (0.00-NA)		
		Dominant	A/A	35 (51.5%)	15 (62.5%)	1.00	0.35	108.7
			A/G-G/G	33 (48.5%)	9 (37.5%)	0.64 (0.25-1.65)		
		Recessive	A/A-A/G	65 (95.6%)	24 (100%)	1.00	0.17	107.8
			G/G	3 (4.4%)	0 (0%)	0.00 (0.00-NA)		
		Overdominant	A/A-G/G	38 (55.9%)	15 (62.5%)	1.00	0.57	109.3
			A/G	30 (44.1%)	9 (37.5%)	0.76 (0.29-1.98)		
		Log-additive	---	---	---	0.60 (0.25-1.44)	0.24	108.2
DC-SIGN	rs735240 (G/A)	Codominant	G/G	27 (39.7%)	8 (33.3%)	1.00	0.3	109.2
			G/A	29 (42.6%)	8 (33.3%)	0.93 (0.31-2.83)		
			A/A	12 (17.6%)	8 (33.3%)	2.25 (0.68-7.42)		
		Dominant	G/G	27 (39.7%)	8 (33.3%)	1.00	0.58	109.3
			G/A-A/A	41 (60.3%)	16 (66.7%)	1.32 (0.50-3.50)		
		Recessive	G/G-G/A	56 (82.3%)	16 (66.7%)	1.00	0.12	107.2
			A/A	12 (17.6%)	8 (33.3%)	2.33 (0.81-6.69)		
		Overdominant	G/G-A/A	39 (57.4%)	16 (66.7%)	1.00	0.42	109
			G/A	29 (42.6%)	8 (33.3%)	0.67 (0.25-1.78)		
		Log-additive	---	---	---	1.47 (0.79-2.72)	0.22	108.1
TLR2	rs5743708 (G/A)	---	G/G	61 (89.7%)	21 (87.5%)	1.00	0.77	109.5
			G/A	7 (10.3%)	3 (12.5%)	1.24 (0.29-5.26)		
TLR4	rs4986791 (C/T)	---	C/C	63 (92.7%)	20 (83.3%)	1.00	0.21	108
			C/T	5 (7.3%)	4 (16.7%)	2.52 (0.62-10.30)		
TLR9	rs352140 (C/T)	Codominant	T/T	22 (32.4%)	8 (33.3%)	1.00	0.37	109.6
			C/T	37 (54.4%)	10 (41.7%)	0.74 (0.26-2.16)		
			C/C	9 (13.2%)	6 (25%)	1.83 (0.49-6.81)		
		Dominant	T/T	22 (32.4%)	8 (33.3%)	1.00	0.93	109.6
			C/T-C/C	46 (67.7%)	16 (66.7%)	0.96 (0.36-2.57)		
		Recessive	T/T-C/T	59 (86.8%)	18 (75%)	1.00	0.2	107.9
			C/C	9 (13.2%)	6 (25%)	2.19 (0.69-6.97)		

Overdominant	T/T-C/C	31 (45.6%)	14 (58.3%)	1.00	0.28	108.5
	C/T	37 (54.4%)	10 (41.7%)	0.60 (0.23-1.53)		
Log-additive	---	---	---	1.26 (0.64-2.50)	0.5	109.2

Data presented as number (%), cUS, cranial ultrasound; cCMV, congenital HCMV infection; OR, odds ratio; CI, confidence interval; NA, not applicable; AIC, Akaike information criteria; IL, Interleukin; CCL 2, C-C motif chemokine ligand 2; DC-SIGN, dendritic cell-specific ICAM-grabbing non-integrin; TLR, Toll-like receptor; [1] SNP database (dbSNP) reference number (ID number); [2] P-value for comparison between asymptomatic and symptomatic group.

Table S3. Associations between SNPs and calcifications on cUS in infants with cCMV.

Gene	dbSNP ID number[1]	Genetic Model	Genotype	Without calcifications *n* = 67	Calcifications *n* = 25	OR (95% CI)	*P*-value[2]	AIC
IL1B	rs16944 (G/A)	Codominant	G/G	26 (38.8%)	10 (40%)	1.00	0.99	113.6
			G/A	36 (53.7%)	13 (52%)	0.94 (0.36-2.47)		
			A/A	5 (7.5%)	2 (8%)	1.04 (0.17-6.26)		
		Dominant	G/G	26 (38.8%)	10 (40%)	1.00	0.92	111.6
			G/A-A/A	41 (61.2%)	15 (60%)	0.95 (0.37-2.43)		
		Recessive	G/G-G/A	62 (92.5%)	23 (92%)	1.00	0.93	111.6
			A/A	5 (7.5%)	2 (8%)	1.08 (0.20-5.95)		
		Overdominant	G/G-A/A	31 (46.3%)	12 (48%)	1.00	0.88	111.6
			G/A	36 (53.7%)	13 (52%)	0.93 (0.37-2.34)		
		Log-additive	---	---	---	0.98 (0.46-2.10)	0.96	111.6
IL12B	rs3212227 (T/G)	Codominant	T/T	38 (56.7%)	19 (76%)	1.00	0.15	109.9
			T/G	24 (35.8%)	4 (16%)	0.33 (0.10-1.10)		
			G/G	5 (7.5%)	2 (8%)	0.80 (0.14-4.51)		
		Dominant	T/T	38 (56.7%)	19 (76%)	1.00	0.083	108.6
			T/G-G/G	29 (43.3%)	6 (24%)	0.41 (0.15-1.17)		
		Recessive	T/T-T/G	62 (92.5%)	23 (92%)	1.00	0.93	111.6
			G/G	5 (7.5%)	2 (8%)	1.08 (0.20-5.95)		
		Overdominant	T/T-G/G	43 (64.2%)	21 (84%)	1.00	0.056	108
			T/G	24 (35.8%)	4 (16%)	0.34 (0.10-1.11)		
		Log-additive	---	---	---	0.60 (0.26-1.34)	0.19	109.9
IL28B	rs12979860 (C/T)	Codominant	C/C	29 (43.3%)	12 (48%)	1.00	0.81	113.2
			T/C	29 (43.3%)	9 (36%)	0.75 (0.27-2.05)		
			T/T	9 (13.4%)	4 (16%)	1.07 (0.28-4.17)		
		Dominant	C/C	29 (43.3%)	12 (48%)	1.00	0.69	111.5
			C/T-T/T	38 (56.7%)	13 (52%)	0.83 (0.33-2.08)		
		Recessive	C/C-C/T	58 (86.6%)	21 (84%)	1.00	0.76	111.5
			T/T	9 (13.4%)	4 (16%)	1.23 (0.34-4.41)		
		Overdominant	C/C-T/T	38 (56.7%)	16 (64%)	1.00	0.53	111.2
			C/T	29 (43.3%)	9 (36%)	0.74 (0.29-1.90)		
		Log-additive	---	---	---	0.96 (0.50-1.84)	0.9	111.6
CCL2	rs1024611 (A/G)	Codominant	A/A	34 (50.8%)	16 (64%)	1.00	0.24	110.8
			A/G	30 (44.8%)	9 (36%)	0.64 (0.25-1.65)		
			G/G	3 (4.5%)	0 (0%)	0.00 (0.00-NA)		
		Dominant	A/A	34 (50.8%)	16 (64%)	1.00	0.25	110.3
			A/G-G/G	33 (49.2%)	9 (36%)	0.58 (0.22-1.49)		

		Recessive	A/A-A/G	64 (95.5%)	25 (100%)	1.00	0.16	109.7
			G/G	3 (4.5%)	0 (0%)	0.00 (0.00-NA)		
		Overdominant	A/A-G/G	37 (55.2%)	16 (64%)	1.00	0.45	111.1
		Log-additive	---	---	---	0.55 (0.23-1.32)	0.17	109.7
DC-SIGN	rs735240 (G/A)	Codominant	G/G	22 (32.8%)	13 (52%)	1.00	0.25	110.8
			G/A	29 (43.3%)	8 (32%)	0.47 (0.16-1.32)		
			A/A	16 (23.9%)	4 (16%)	0.42 (0.12-1.54)		
		Dominant	G/G	22 (32.8%)	13 (52%)	1.00	0.095	108.9
			G/A-A/A	45 (67.2%)	12 (48%)	0.45 (0.18-1.15)		
		Recessive	G/G-G/A	51 (76.1%)	21 (84%)	1.00	0.4	110.9
			A/A	16 (23.9%)	4 (16%)	0.61 (0.18-2.03)		
		Overdominant	G/G-A/A	38 (56.7%)	17 (68%)	1.00	0.32	110.7
			G/A	29 (43.3%)	8 (32%)	0.62 (0.23-1.63)		
		Log-additive	---	---	---	0.61 (0.32-1.16)	0.12	109.2
TLR2	rs5743708 (G/A)	---	G/G	62 (92.5%)	20 (80%)	1.00	0.1	109
			G/A	5 (7.5%)	5 (20%)	3.10 (0.81-11.82)		
TLR4	rs4986791 (C/T)	---	C/C	61 (91%)	22 (88%)	1.00	0.67	111.5
			C/T	6 (9%)	3 (12%)	1.39 (0.32-6.02)		
TLR9	rs352140 (C/T)	Codominant	T/T	21 (31.3%)	9 (36%)	1.00	0.76	113.1
			C/T	34 (50.8%)	13 (52%)	0.89 (0.33-2.45)		
			C/C	12 (17.9%)	3 (12%)	0.58 (0.13-2.58)		
		Dominant	T/T	21 (31.3%)	9 (36%)	1.00	0.67	111.5
			C/T-C/C	46 (68.7%)	16 (64%)	0.81 (0.31-2.13)		
		Recessive	T/T-C/T	55 (82.1%)	22 (88%)	1.00	0.48	111.1
			C/C	12 (17.9%)	3 (12%)	0.62 (0.16-2.43)		
		Overdominant	T/T-C/C	33 (49.2%)	12 (48%)	1.00	0.91	111.6
			C/T	34 (50.8%)	13 (52%)	1.05 (0.42-2.64)		
		Log-additive	---	---	---	0.79 (0.40-1.57)	0.51	111.2

Data presented as number (%), cUS, cranial ultrasound; cCMV, congenital HCMV infection; OR, odds ratio; CI, confidence interval; NA, not applicable; AIC, Akaike information criteria; IL, Interleukin; CCL 2, C-C motif chemokine ligand 2; DC-SIGN, dendritic cell-specific ICAM-grabbing non-integrin; TLR, Toll-like receptor; [1] SNP database (dbSNP) reference number (ID number); [2] P-value for comparison between asymptomatic and symptomatic group.

Table S4. Associations between SNPs and abnormal myelination on MRI in infants with cCMV.

Gene	dbSNP ID number[1]	Genetic Model	Genotype	Without abnormal myelination *n* = 72	Abnormal myelination *n* = 15	OR (95% CI)	*P*-value[2]	AIC
IL1B	rs16944 (G/A)	Codominant	G/G	27 (37.5%)	8 (53.3%)	1.00	0.18	82.5
			G/A	38 (52.8%)	7 (46.7%)	0.62 (0.20-1.92)		
			A/A	7 (9.7%)	0 (0%)	0.00 (0.00-NA)		
		Dominant	G/G	27 (37.5%)	8 (53.3%)	1.00	0.26	82.7
			G/A-A/A	45 (62.5%)	7 (46.7%)	0.52 (0.17-1.61)		
		Recessive	G/G-G/A	65 (90.3%)	15 (100%)	1.00	0.096	81.2
			A/A	7 (9.7%)	0 (0%)	0.00 (0.00-NA)		
		Overdominant	G/G-A/A	34 (47.2%)	8 (53.3%)	1.00	0.67	83.8
			G/A	38 (52.8%)	7 (46.7%)	0.78 (0.26-2.39)		
		Log-additive	-	-	-	0.48 (0.18-1.30)	0.13	81.7

IL12B	rs3212227 (T/G)	Codominant	T/T	41 (56.9%)	12 (80%)	1.00	0.11	81.5
			T/G	24 (33.3%)	3 (20%)	0.43 (0.11-1.67)		
			G/G	7 (9.7%)	0 (0%)	0.00 (0.00-NA)		
		Dominant	T/T	41 (56.9%)	12 (80%)	1.00	0.084	81
			T/G-G/G	31 (43.1%)	3 (20%)	0.33 (0.09-1.27)		
		Recessive	T/T-T/G	65 (90.3%)	15 (100%)	1.00	0.096	81.2
			G/G	7 (9.7%)	0 (0%)	0.00 (0.00-NA)		
		Overdominant	T/T-G/G	48 (66.7%)	12 (80%)	1.00	0.29	82.9
			T/G	24 (33.3%)	3 (20%)	0.50 (0.13-1.94)		
		Log-additive	-	-	-	0.34 (0.10-1.16)	0.049	80.1
IL28B	rs12979860 (C/T)	Codominant	C/C	30 (41.7%)	7 (46.7%)	1.00	0.69	85.2
			C/T	32 (44.4%)	5 (33.3%)	0.67 (0.19-2.34)		
			T/T	10 (13.9%)	3 (20%)	1.29 (0.28-5.94)		
		Dominant	C/C	30 (41.7%)	7 (46.7%)	1.00	0.72	83.9
			C/T-T/T	42 (58.3%)	8 (53.3%)	0.82 (0.27-2.50)		
		Recessive	C/C-C/T	62 (86.1%)	12 (80%)	1.00	0.56	83.6
			T/T	10 (13.9%)	3 (20%)	1.55 (0.37-6.48)		
		Overdominant	C/C-T/T	40 (55.6%)	10 (66.7%)	1.00	0.42	83.3
			C/T	32 (44.4%)	5 (33.3%)	0.63 (0.19-2.01)		
		Log-additive	-	-	-	1.02 (0.47-2.24)	0.96	84
CCL2	rs1024611 (A/G)	Codominant	A/A	37 (51.4%)	8 (53.3%)	1.00	0.76	85.4
			A/G	33 (45.8%)	6 (40%)	0.84 (0.26-2.68)		
			G/G	2 (2.8%)	1 (6.7%)	2.31 (0.19-28.72)		
		Dominant	A/A	37 (51.4%)	8 (53.3%)	1.00	0.89	84
			A/G-G/G	35 (48.6%)	7 (46.7%)	0.92 (0.30-2.82)		
		Recessive	A/A-A/G	70 (97.2%)	14 (93.3%)	1.00	0.49	83.5
			G/G	2 (2.8%)	1 (6.7%)	2.50 (0.21-29.50)		
		Overdominant	A/A-G/G	39 (54.2%)	9 (60%)	1.00	0.68	83.8
			A/G	33 (45.8%)	6 (40%)	0.79 (0.25-2.44)		
		Log-additive	-	-	-	1.06 (0.40-2.83)	0.9	84
DC-SIGN	rs735240 (G/A)	Codominant	G/G	26 (36.1%)	5 (33.3%)	1.00	0.93	85.8
			G/A	30 (41.7%)	6 (40%)	1.04 (0.28-3.81)		
			A/A	16 (22.2%)	4 (26.7%)	1.30 (0.30-5.57)		
		Dominant	G/G	26 (36.1%)	5 (33.3%)	1.00	0.84	83.9
			G/A-A/A	46 (63.9%)	10 (66.7%)	1.13 (0.35-3.67)		
		Recessive	G/G-G/A	56 (77.8%)	11 (73.3%)	1.00	0.71	83.9
			A/A	16 (22.2%)	4 (26.7%)	1.27 (0.36-4.54)		
		Overdominant	G/G-A/A	42 (58.3%)	9 (60%)	1.00	0.9	84
			G/A	30 (41.7%)	6 (40%)	0.93 (0.30-2.90)		
		Log-additive	-	-	-	1.13 (0.54-2.36)	0.74	83.9
TLR2	rs5743708 (G/A)	-	G/G	64 (88.9%)	14 (93.3%)	1.00	0.59	83.7
			G/A	8 (11.1%)	1 (6.7%)	0.57 (0.07-4.94)		
TLR4	rs4986791 (C/T)	-	C/C	67 (93.1%)	12 (80%)	1.00	0.15	81.9
			C/T	5 (6.9%)	3 (20%)	3.35 (0.71-15.90)		
TLR9	rs352140 (C/T)	Codominant	T/T	23 (31.9%)	5 (33.3%)	1.00	0.93	85.8
			T/C	37 (51.4%)	7 (46.7%)	0.87 (0.25-3.07)		
			C/C	12 (16.7%)	3 (20%)	1.15 (0.23-5.65)		
		Dominant	T/T	23 (31.9%)	5 (33.3%)	1.00	0.92	84
			C/T-C/C	49 (68.1%)	10 (66.7%)	0.94 (0.29-3.06)		
		Recessive	T/T-C/T	60 (83.3%)	12 (80%)	1.00	0.76	83.9

			C/C	12 (16.7%)	3 (20%)	1.25 (0.31-5.11)		
		Overdominant	T/T-C/C	35 (48.6%)	8 (53.3%)	1.00	0.74	83.9
			C/T	37 (51.4%)	7 (46.7%)	0.83 (0.27-2.52)		
		Log-additive	-	-	-	1.04 (0.46-2.34)	0.92	84

Data presented as number (%);MRI, magnetic resonance imaging; cCMV, congenital HCMV infection; OR, odds ratio; CI, confidence interval; NA, not applicable; AIC, Akaike information criteria; IL, Interleukin; CCL 2, C-C motif chemokine ligand 2; DC-SIGN, dendritic cell-specific ICAM-grabbing non-integrin; TLR, Toll-like receptor; [1] SNP database (dbSNP) reference number (ID number); [2] P-value for comparison between asymptomatic and symptomatic group.

Table S5. Associations between SNPs and abnormal white matter on MRI in infants with cCMV.

Gene	dbSNP ID number[1]	Genetic Model	Genotype	Without abnormal white matter *n* = 23	Abnormal white matter *n* = 64	OR (95% CI)	*P*-value[2]	AIC
IL1B	rs16944 (G/A)	Codominant	G/G	10 (43.5%)	25 (39.1%)	1.00	0.5	105.1
			G/A	10 (43.5%)	35 (54.7%)	1.40 (0.51-3.87)		
			A/A	3 (13%)	4 (6.2%)	0.53 (0.10-2.82)		
		Dominant	G/G	10 (43.5%)	25 (39.1%)	1.00	0.71	104.4
			G/A-A/A	13 (56.5%)	39 (60.9%)	1.20 (0.46-3.15)		
		Recessive	G/G-G/A	20 (87%)	60 (93.8%)	1.00	0.33	103.5
			A/A	3 (13%)	4 (6.2%)	0.44 (0.09-2.16)		
		Overdominant	G/G-A/A	13 (56.5%)	29 (45.3%)	1.00		
			G/A	10 (43.5%)	35 (54.7%)	1.57 (0.60-4.10)	0.36	103.6
		Log-additive	---	---	---	0.94 (0.43-2.03)	0.87	104.5
IL12B	rs3212227 (T/G)	Codominant	T/T	15 (65.2%)	38 (59.4%)	1.00	0.83	106.1
			T/G	6 (26.1%)	21 (32.8%)	1.38 (0.47-4.09)		
			G/G	2 (8.7%)	5 (7.8%)	0.99 (0.17-5.65)		
		Dominant	T/T	15 (65.2%)	38 (59.4%)	1.00	0.62	104.3
			T/G-G/G	8 (34.8%)	26 (40.6%)	1.28 (0.48-3.46)		
		Recessive	T/T-T/G	21 (91.3%)	59 (92.2%)	1.00	0.89	104.5
			G/G	2 (8.7%)	5 (7.8%)	0.89 (0.16-4.94)		
		Overdominant	T/T-G/G	17 (73.9%)	43 (67.2%)	1.00	0.55	104.1
			T/G	6 (26.1%)	21 (32.8%)	1.38 (0.48-4.02)		
		Log-additive	---	---	---	1.13 (0.53-2.42)	0.75	104.4
IL28B	rs12979860 (C/T)	Codominant	C/C	11 (47.8%)	26 (40.6%)	1.00	0.57	105.4
			C/T	10 (43.5%)	27 (42.2%)	1.14 (0.42-3.14)		
			T/T	2 (8.7%)	11 (17.2%)	2.33 (0.44-12.28)		
		Dominant	C/C	11 (47.8%)	26 (40.6%)	1.00	0.55	104.1
			C/T-T/T	12 (52.2%)	38 (59.4%)	1.34 (0.51-3.49)		
		Recessive	C/C-C/T	21 (91.3%)	53 (82.8%)	1.00	0.3	103.4
			T/T	2 (8.7%)	11 (17.2%)	2.18 (0.44-10.68)		
		Overdominant	C/C-T/T	13 (56.5%)	37 (57.8%)	1.00	0.91	104.5
		Log-additive	---	---	---	1.38 (0.69-2.79)	0.36	103.6
CCL2	rs1024611 (A/G)	Codominant	A/A	13 (56.5%)	32 (50%)	1.00	0.8	106.1
			A/G	9 (39.1%)	30 (46.9%)	1.35 (0.51-3.63)		
			G/G	1 (4.3%)	2 (3.1%)	0.81 (0.07-9.76)		
		Dominant	A/A	13 (56.5%)	32 (50%)	1.00	0.59	104.2
			A/G-G/G	10 (43.5%)	32 (50%)	1.30 (0.50-3.39)		

		Recessive	A/A-A/G	22 (95.7%)	62 (96.9%)	1.00	0.79	104.4
			G/G	1 (4.3%)	2 (3.1%)	0.71 (0.06-8.22)		
		Overdominant	A/A-G/G	14 (60.9%)	34 (53.1%)	1.00	0.52	104.1
			A/G	9 (39.1%)	30 (46.9%)	1.37 (0.52-3.62)		
		Log-additive	---	---	---	1.18 (0.50-2.78)	0.7	104.3
DC-SIGN	rs735240 (G/A)	Codominant	G/G	8 (34.8%)	23 (35.9%)	1.00	0.68	105.7
			G/A	11 (47.8%)	25 (39.1%)	0.79 (0.27-2.31)		
			A/A	4 (17.4%)	16 (25%)	1.39 (0.36-5.42)		
		Dominant	G/G	8 (34.8%)	23 (35.9%)	1.00	0.92	104.5
			G/A-A/A	15 (65.2%)	41 (64.1%)	0.95 (0.35-2.58)		
		Recessive	G/G-G/A	19 (82.6%)	48 (75%)	1.00	0.45	103.9
			A/A	4 (17.4%)	16 (25%)	1.58 (0.47-5.35)		
		Overdominant	G/G-A/A	12 (52.2%)	39 (60.9%)	1.00	0.47	104
			G/A	11 (47.8%)	25 (39.1%)	0.70 (0.27-1.83)		
		Log-additive	---	---	---	1.12 (0.59-2.11)	0.72	104.4
TLR2	rs5743708 (G/A)	---	G/G	21 (91.3%)	57 (89.1%)	1.00	0.76	104.4
			G/A	2 (8.7%)	7 (10.9%)	1.29 (0.25-6.71)		
TLR4	rs4986791 (C/T)	---	C/C	21 (91.3%)	58 (90.6%)	1.00	0.92	104.5
			C/T	2 (8.7%)	6 (9.4%)	1.09 (0.20-5.81)		
TLR9	rs352140 (C/T)	Codominant	T/T	8 (34.8%)	20 (31.2%)	1.00	0.41	104.7
			C/T	13 (56.5%)	31 (48.4%)	0.95 (0.34-2.71)		
			C/C	2 (8.7%)	13 (20.3%)	2.60 (0.48-14.23)		
		Dominant	T/T	8 (34.8%)	20 (31.2%)	1.00	0.76	104.4
			C/T-C/C	15 (65.2%)	44 (68.8%)	1.17 (0.43-3.21)		
		Recessive	T/T-C/T	21 (91.3%)	51 (79.7%)	1.00	0.18	102.7
			C/C	2 (8.7%)	13 (20.3%)	2.68 (0.56-12.90)		
		Overdominant	T/T-C/C	10 (43.5%)	33 (51.6%)	1.00	0.51	104.1
			C/T	13 (56.5%)	31 (48.4%)	0.72 (0.28-1.89)		
		Log-additive	---	---	---	1.39 (0.68-2.82)	0.36	103.7

Data presented as number (%); MRI, magnetic resonance imaging; cCMV, congenital HCMV infection; OR, odds ratio; CI, confidence interval; NA, not applicable; AIC, Akaike information criteria; IL, Interleukin; CCL 2, C-C motif chemokine ligand 2; DC-SIGN, dendritic cell-specific ICAM-grabbing non-integrin; TLR, Toll-like receptor; [1] SNP database (dbSNP) reference number (ID number); [2] P-value for comparison between asymptomatic and symptomatic group.

Table S6. Associations between SNPs and calcifications on MRI in infants with cCMV.

Gene	dbSNP ID number[1]	Genetic Model	Genotype	Without calcification *n* = 72	Calcification *n* = 15	OR (95% CI)	*P*-value[2]	AIC
IL1B	rs16944 (G/A)	Codominant	G/G	29 (40.3%)	6 (40%)	1.00	0.97	85.9
			G/A	37 (51.4%)	8 (53.3%)	1.05 (0.33-3.35)		
			A/A	6 (8.3%)	1(6.7%)	0.81 (0.08-7.97)		
		Dominant	G/G	29 (40.3%)	6 (40%)	1.00	0.98	84
			G/A-A/A	43 (59.7%)	9 (60%)	1.01 (0.33-3.15)		
		Recessive	G/G-G/A	66 (91.7%)	14(93.3%)	1.00	0.83	83.9
			A/A	6 (8.3%)	1(6.7%)	0.79 (0.09-7.05)		
		Overdominant	G/G-A/A	35 (48.6%)	7 (46.7%)	1.00	0.89	84
			G/A	37 (51.4%)	8 (53.3%)	1.08 (0.35-3.30)		
		Log-additive	---	---	---	0.96 (0.39-2.38)	0.94	84
IL12B		Codominant	T/T	41 (56.9%)	12 (80%)	1.00	0.11	81.5

			T/G	24 (33.3%)	3 (20%)	0.43 (0.11-1.67)		
			G/G	7 (9.7%)	0 (0%)	0.00 (0.00-NA)		
	rs3212227 (T/G)	Dominant	T/T	41 (56.9%)	12 (80%)	1.00	0.084	81
			T/G-G/G	31 (43.1%)	3 (20%)	0.33 (0.09-1.27)		
		Recessive	T/T-T/G	65 (90.3%)	15 (100%)	1.00	0.096	81.2
			G/G	7 (9.7%)	0 (0%)	0.00 (0.00-NA)		
		Overdominant	T/T-G/G	48 (66.7%)	12 (80%)	1.00	0.29	82.9
			T/G	24 (33.3%)	3 (20%)	0.50 (0.13-1.94)		
		Log-additive	---	---	---	0.34 (0.10-1.16)	0.049	80.1
IL28B	rs12979860 (C/T)	Codominant	C/C	30 (41.7%)	7 (46.7%)	1.00	0.69	85.2
			C/T	32 (44.4%)	5 (33.3%)	0.67 (0.19-2.34)		
			T/T	10 (13.9%)	3 (20%)	1.29 (0.28-5.94)		
		Dominant	C/C	30 (41.7%)	7 (46.7%)	1.00	0.72	83.9
			C/T-T/T	42 (58.3%)	8 (53.3%)	0.82 (0.27-2.50)		
		Recessive	C/C-C/T	62 (86.1%)	12 (80%)	1.00	0.56	83.6
			T/T	10 (13.9%)	3 (20%)	1.55 (0.37-6.48)		
		Overdominant	C/C-T/T	40 (55.6%)	10 (66.7%)	1.00	0.42	83.3
			C/T	32 (44.4%)	5 (33.3%)	0.63 (0.19-2.01)		
		Log-additive	---	---	---	1.02 (0.47-2.24)	0.96	84
CCL2	rs1024611 (A/G)	Codominant	A/A	37 (51.4%)	8 (53.3%)	1.00	0.56	84.8
			A/G	32 (44.4%)	7 (46.7%)	1.01 (0.33-3.10)		
			G/G	3 (4.2%)	0 (0%)	0.00 (0.00-NA)		
		Dominant	A/A	37 (51.4%)	8 (53.3%)	1.00	0.89	84
			A/G-G/G	35 (48.6%)	7 (46.7%)	0.92 (0.30-2.82)		
		Recessive	A/A-A/G	69 (95.8%)	15 (100%)	1.00	0.28	82.8
			G/G	3 (4.2%)	0 (0%)	0.00 (0.00-NA)		
		Overdominant	A/A-G/G	40 (55.6%)	8 (53.3%)	1.00	0.88	84
			A/G	32 (44.4%)	7 (46.7%)	1.09 (0.36-3.34)		
		Log-additive	---	---	---	0.82 (0.30-2.25)	0.7	83.8
DC-SIGN	rs735240 (G/A)	Codominant	G/G	27 (37.5%)	4 (26.7%)	1.00	0.58	84.9
			G/A	28 (38.9%)	8 (53.3%)	1.93 (0.52-7.16)		
			A/A	17 (23.6%)	3 (20%)	1.19 (0.24-5.99)		
		Dominant	G/G	27 (37.5%)	4 (26.7%)	1.00	0.42	83.3
			G/A-A/A	45 (62.5%)	11 (73.3%)	1.65 (0.48-5.70)		
		Recessive	G/G-G/A	55 (76.4%)	12 (80%)	1.00	0.76	83.9
			A/A	17 (23.6%)	3 (20%)	0.81 (0.20-3.21)		
		Overdominant	G/G-A/A	44 (61.1%)	7 (46.7%)	1.00	0.3	82.9
			G/A	28 (38.9%)	8 (53.3%)	1.80 (0.59-5.50)		
		Log-additive	---	---	---	1.13 (0.54-2.36)	0.74	83.9
TLR2	rs5743708 (G/A)	---	G/G	65 (90.3%)	13 (86.7%)	1.00	0.69	83.8
			G/A	7 (9.7%)	2 (13.3%)	1.43 (0.27-7.67)		
TLR4	rs4986791 (C/T)	---	C/C	66 (91.7%)	13 (86.7%)	1.00	0.56	83.6
			C/T	6 (8.3%)	2 (13.3%)	1.69 (0.31-9.33)		
TLR9	rs352140 (C/T)	Codominant	T/T	23 (31.9%)	5 (33.3%)	1.00	0.41	84.2
			C/T	35 (48.6%)	9 (60%)	1.18 (0.35-3.98)		
			C/C	14 (19.4%)	1 (6.7%)	0.33 (0.03-3.11)		
		Dominant	T/T	23 (31.9%)	5 (33.3%)	1.00	0.92	84
			C/T-C/C	49 (68.1%)	10 (66.7%)	0.94 (0.29-3.06)		
		Recessive	T/T-C/T	58 (80.6%)	14 (93.3%)	1.00	0.19	82.3

	C/C	14 (19.4%)	1 (6.7%)	0.30 (0.04-2.44)		
Overdominant	T/T-C/C	37 (51.4%)	6 (40%)	1.00	0.42	83.3
	C/T	35 (48.6%)	9 (60%)	1.59 (0.51-4.92)		
Log-additive	---	---	---	0.74 (0.32-1.69)	0.46	83.5

Data presented as number (%); MRI, magnetic resonance imaging; cCMV, congenital HCMV infection; OR, odds ratio; CI, confidence interval; NA, not applicable; AIC, Akaike information criteria; IL, Interleukin; CCL 2, C-C motif chemokine ligand 2; DC-SIGN, dendritic cell-specific ICAM-grabbing non-integrin; TLR, Toll-like receptor; [1] SNP database (dbSNP) reference number (ID number); [2] P-value for comparison between asymptomatic and symptomatic group.

Table S7. Associations between SNPs and cortical migration defects on MRI in infants with cCMV.

Gene	dbSNP ID number[1]	Genetic Model	Genotype	Without cortical migration defects *n* = 75	Cortical migration defects *n* = 12	OR (95% CI)	*P*-value[2]	AIC
IL1B	rs16944 (G/A)	Codominant	G/G	28 (37.3%)	7 (58.3%)	1.00	0.36	73.8
			G/A	41 (54.7%)	4 (33.3%)	0.39 (0.10-1.46)		
			A/A	6 (8%)	1 (8.3%)	0.67 (0.07-6.47)		
		Dominant	G/G	28 (37.3%)	7 (58.3%)	1.00	0.17	71.9
			G/A-A/A	47 (62.7%)	5 (41.7%)	0.43 (0.12-1.47)		
		Recessive	G/G-G/A	69 (92%)	11 (91.7%)	1.00	0.97	73.8
			A/A	6 (8%)	1 (8.3%)	1.05 (0.11-9.54)		
		Overdominant	G/G-A/A	34 (45.3%)	8 (66.7%)	1.00	0.17	71.9
			G/A	41 (54.7%)	4 (33.3%)	0.41 (0.11-1.50)		
		Log-additive	---	---	---	0.56 (0.19-1.62)	0.27	72.6
IL12B	rs3212227 (T/G)	Codominant	T/T	46 (61.3%)	7 (58.3%)	1.00	0.28	73.2
			T/G	22 (29.3%)	5 (41.7%)	1.49 (0.43-5.24)		
			G/G	7 (9.3%)	0 (0%)	0.00 (0.00-NA)		
		Dominant	T/T	46 (61.3%)	7 (58.3%)	1.00	0.84	73.8
			T/G-G/G	29 (38.7%)	5 (41.7%)	1.13 (0.33-3.91)		
		Recessive	T/T-T/G	68 (90.7%)	12 (100%)	1.00	0.14	71.6
			G/G	7 (9.3%)	0 (0%)	0.00 (0.00-NA)		
		Overdominant	T/T-G/G	53 (70.7%)	7 (58.3%)	1.00	0.4	73.1
			T/G	22 (29.3%)	5 (41.7%)	1.72 (0.49-6.01)		
		Log-additive	---	---	---	0.85 (0.32-2.30)	0.75	73.7
IL28B	rs12979860 (C/T)	Codominant	C/C	31 (41.3%)	6 (50%)	1.00	0.78	75.3
			C/T	33 (44%)	4 (33.3%)	0.63 (0.16-2.43)		
			T/T	11 (14.7%)	2 (16.7%)	0.94 (0.16-5.36)		
		Dominant	C/C	31 (41.3%)	6 (50%)	1.00	0.57	73.5
			C/T-T/T	44 (58.7%)	6 (50%)	0.70 (0.21-2.39)		
		Recessive	C/C-C/T	64 (85.3%)	10 (83.3%)	1.00	0.86	73.8
			T/T	11 (14.7%)	2 (16.7%)	1.16 (0.22-6.04)		
		Overdominant	C/C-T/T	42 (56%)	8 (66.7%)	1.00	0.48	73.3
			C/T	33 (44%)	4 (33.3%)	0.64 (0.18-2.30)		
		Log-additive	---	---	---	0.87 (0.36-2.10)	0.76	73.7
CCL2	rs1024611 (A/G)	Codominant	A/A	39 (52%)	6 (50%)	1.00	0.61	74.8
			A/G	33 (44%)	6 (50%)	1.18 (0.35-4.01)		
			G/G	3 (4%)	0 (0%)	0.00 (0.00-NA)		

		Dominant	A/A	39 (52%)	6 (50%)	1.00	0.9	73.8
			A/G-G/G	36 (48%)	6 (50%)	1.08 (0.32-3.67)		
		Recessive	A/A-A/G	72 (96%)	12 (100%)	1.00	0.34	72.9
			G/G	3 (4%)	0 (0%)	0.00 (0.00-NA)		
		Overdominant	A/A-G/G	42 (56%)	6 (50%)	1.00	0.7	73.7
			A/G	33 (44%)	6 (50%)	1.27 (0.38-4.31)		
		Log-additive	---	---	---	0.94 (0.32-2.78)	0.91	73.8
DC-SIGN	rs735240 (G/A)	Codominant	G/G	29 (38.7%)	2 (16.7%)	1.00	0.15	72
			G/A	28 (37.3%)	8 (66.7%)	4.14 (0.81-21.23)		
			A/A	18 (24%)	2 (16.7%)	1.61 (0.21-12.47)		
		Dominant	G/G	29 (38.7%)	2 (16.7%)	1.00	0.12	71.4
			G/A-A/A	46 (61.3%)	10 (83.3%)	3.15 (0.64-15.42)		
		Recessive	G/G-G/A	57 (76%)	10 (83.3%)	1.00	0.56	73.5
			A/A	18 (24%)	2 (16.7%)	0.63 (0.13-3.16)		
		Overdominant	G/G-A/A	47 (62.7%)	4 (33.3%)	1.00	0.057	70.2
			G/A	28 (37.3%)	8 (66.7%)	3.36 (0.93-12.17)		
		Log-additive	---	---	---	1.29 (0.58-2.88)	0.53	73.4
TLR2	rs5743708 (G/A)	---	G/G	69 (92%)	9 (75%)	1.00	0.11	71.2
			G/A	6 (8%)	3 (25%)	3.83 (0.81-18.07)		
TLR4	rs4986791 (C/T)	---	C/C	67 (89.3%)	12 (100%)	1.00	0.11	71.3
			C/T	8 (10.7%)	0 (0%)	0.00 (0.00-NA)		
TLR9	rs352140 (C/T)	Codominant	T/T	24 (32%)	4 (33.3%)	1.00	0.63	74.9
			C/T	37 (49.3%)	7 (58.3%)	1.14 (0.30-4.30)		
			C/C	14 (18.7%)	1 (8.3%)	0.43 (0.04-4.23)		
		Dominant	T/T	24 (32%)	4 (33.3%)	1.00	0.93	73.8
			C/T -C/C	51 (68%)	8 (66.7%)	0.94 (0.26-3.43)		
		Recessive	T/T-C/T	61 (81.3%)	11 (91.7%)	1.00	0.34	72.9
			C/C	14 (18.7%)	1 (8.3%)	0.40 (0.05-3.33)		
		Overdominant	T/T-C/C	38 (50.7%)	5 (41.7%)	1.00	0.56	73.5
			C/T	37 (49.3%)	7 (58.3%)	1.44 (0.42-4.94)		
		Log-additive	---	---	---	0.78 (0.31-1.92)	0.58	73.5

Data presented as number (%); MRI, magnetic resonance imaging; cCMV, congenital HCMV infection; OR, odds ratio; CI, confidence interval; NA, not applicable; AIC, Akaike information criteria; IL, Interleukin; CCL 2, C-C motif chemokine ligand 2; DC-SIGN, dendritic cell-specific ICAM-grabbing non-integrin; TLR, Toll-like receptor; [1] SNP database (dbSNP) reference number (ID number); [2] P-value for comparison between asymptomatic and symptomatic group.
